# Efficacy and safety of acupuncture in the treatment of stroke complicated with sleep apnea syndrome: A systematic review and meta-analysis of randomized controlled trials

**DOI:** 10.1097/MD.0000000000033241

**Published:** 2023-04-14

**Authors:** Huanyu Gao, Zunqi Kan, Yuqing Fang, Ning Wang, Wenli Yan, Mengqi Yang, Yongmei Song

**Affiliations:** a Institute of Literature and Culture of Traditional Chinese Medicine, Shandong University of Traditional Chinese Medicine, Jinan, Shandong, China; b College of Traditional Chinese Medicine, Shandong University of Traditional Chinese Medicine, Jinan, Shandong, China; c College of the First Clinical Medical, Shandong University of Traditional Chinese Medicine, Jinan, Shandong, China.

**Keywords:** acupuncture, meta-analysis, sleep apnea syndrome, stroke, systematic review

## Abstract

stroke patients often have a combination of sleep apnea syndrome, which is an important and modifiable risk factor for stroke prognosis. Acupuncture is one of the measures for sleep apnea syndrome, and it is also widely used in stroke. However, we are concerned that its efficacy and safety in the treatment of stroke with sleep apnea syndrome are not yet clear.

**Methods:**

This systematic review and meta-analysis was performed in accordance with the Preferred Reporting Items for Systematic Reviews and Meta-Analyses schema and was registered with INPLASY (registration number: INPLASY202250113). The following 8 databases were searched: PubMed, Cochrane Library (CENTRAL), Embase, Web of Science, China National Knowledge Infrastructure, Chongqing VIP Information, WanFang Data, and China Biomedical Literature Database limited from the establishment of each database to May 4, 2022. Subject headings, free words, and keywords were used for retrieval. Relevant literature was supplemented by consulting other resources. We assessed the risk of bias in the included studies using the Cochrane risk of bias tool. RevMan 5.4 software (The Cochrane Collaboration, 2020) was used to perform the meta-analysis.

**Results:**

Six records were included, including a total of 513 participants: 256 in the experimental group and 257 in the control group. The results showed that the total effective rate (relative risk = 1.23, 95% confidence interval (CI): 1.13, 1.34, *P* < .00001), apnea-hypopnea index (mean difference (MD) = −8.39, 95% CI: −9.19, −7.59, *P* < .00001), Epworth Sleepiness Scale score (MD = −1.59, 95% CI: −2.66, −0.52, *P* = .004), minimal oxygen saturation (MD = 4.99, 95% CI: 3.5, 6.47, *P* < .00001), longest duration of apnea (MD = −7.47, 95% CI: −8.97, −5.97, *P* < .00001), longest duration of apnea (MD = −6.48, 95% CI: −8.60, −4.35, *P* < .00001), and S100β levels (standard mean difference = −1.52, 95% CI: −1.87, −1.18, *P* < .00001) were better in the experimental group than in the control group. Simultaneously, the effect of reducing the neuron-specific enolase level in the experimental group was comparable to that in the control group (MD = -3.40, 95% CI: −9.08, 2.29, *P* = .24).

**Conclusions:**

Acupuncture can improve the clinical symptoms and related laboratory indicators for sleep apnea syndrome in patients with stroke. More high-quality trials remain urgently needed.

## 1. Introduction

Sleep apnea syndrome (SAs) is a sleep disorder in which breathing stops during sleep. The International Classification of Sleep Disorders (Third Edition) states that sleep-related respiratory disorders include obstructive sleep apnea syndrome (OSAs), central sleep apnea syndrome (CSAs), sleep-related hypopnea disorder, and sleep-related hypoxemia disorder.^[1]^ The most common type is OSAs, characterized by partial or complete collapse of the upper airway during sleep,^[2]^ causing snoring, morning headaches, daytime sleepiness, memory loss, and a series of symptoms. The incidence rate of CSAs is significantly lower than that of OSAs, and it is not related to anatomic obstruction but to insufficient respiratory drive leading to ventilatory disorders. Sleep-related hypoxemia disorder is caused by inadequate sleep ventilation and increased arterial carbon dioxide levels.^[3]^

The diagnosis of SAs is mainly confirmed by polysomnography. The disease can be diagnosed when the apnea-hypopnea index (AHI) is ≥ 5 times/hours, and the higher the AHI, the more serious the illness will be.^[4]^ This disease tends to occur in male, overweight middle-aged, and older people and is usually complicated by cardiovascular and cerebrovascular diseases,^[5]^ making it a central sleep disorder associated with stroke.

Stroke is a major disease of the nervous system, and there is a bidirectional relationship between stroke and SAs.^[6]^ A study reported that the prevalence of SAs after a stroke was nearly 75%.^[7]^ The pathogenesis is still unclear. A few smaller studies have examined the relationship between SAs and infarction location.^[8,9]^ One study suggested that the exacerbation of preexisting SAs risk factors due to stroke was more important than the location.^[10]^ Dysphagia and airway collapse can lead to increased airway resistance, which is a common manifestation after stroke and is directly associated with SAs.^[11]^ Airway obstruction can also occur if the stroke causes severe physical disability, prolonged bed rest, fluid retention in the neck, and increased tissue pressure.^[12]^ Same limb movement leads to the limitation of sleep posture and the increase of supine posture, which becomes one of the reasons for the increased AHI.^[11]^ Moreover, the pathological changes in substance metabolism and vascular endothelium associated with SAs also increase the risk of stroke.^[13]^ It is possible that these factors accelerate the formation of atherosclerosis. Abnormal blood pressure is also a risk factor for stroke, and the blood pressure of SAs patients can be abnormally increased at night.^[14]^ Some studies have suggested that SAs is an important and modifiable risk factor for stroke prognosis.^[15–17]^

Active intervention of SAs will bring high clinical value, but it is a multi-heterogeneous disease that is difficult to treat. To the best of our knowledge, there are no specific drugs for treatment. Currently, the main treatment measures include changing the behavior of patients through health education, adopting device assistance (e.g., continuous positive airway pressure [CPAP]), nerve electrical stimulation therapy, or surgical treatment.

Patients should be encouraged to quit smoking and drinking, lose weight through exercise, avoid sleeping in prone positions, and stop hazardous work when they feel sleepy,^[18]^ which are essential therapeutic processes. Additionally, more robust modalities are required. As first-line therapy, CPAP is of great significance for improving clinical symptoms.^[19]^ However, this approach also has drawbacks, such as poor patient adherence, and difficulty in ensuring efficacy when weaned. Some studies have indicated that the therapeutic effect of CPAP in patients with stroke is not significant. Neither poststroke symptoms nor ambulatory blood pressure levels improved significantly.^[20]^ The cardio-cerebrovascular protective effect of this therapy needs to be clinically verified. Surgery can relieve symptoms by removing excess tissue; however, this is not the first choice for this disease. Postoperatively, patients also face a series of problems, such as postoperative pain, obstruction of eating, taste changes, and tongue dysfunction.^[21]^ Electrical nerve stimulation is a non-anatomical surgical treatment. The opening of the upper airway is prompted by the installation of special equipment, with the hypoglossal nerve as the primary target of electrical stimulation.^[22]^ However, due to the physiological differences in laryngeal shape and muscle contraction degree of different individuals, nerve stimulation therapy only has a good effect on some patients with OSAs.^[23]^ Additionally, it has the disadvantages of being traumatic and costly.

As a vital treatment method in traditional Chinese medicine (TCM), acupuncture has the advantages of being simple, having a considerable curative effect, and having fewer adverse reactions than surgical treatment. A previous study showed that acupuncture at specific acupoints can improve the collapse of throat tissue, thereby alleviating the symptoms of SAs.^[24]^ On the other hand, acupuncture is also widely used in treating stroke. Nevertheless, its efficacy and safety in stroke patients with SAs remain unclear. Wang et al^[25]^ published a meta-analysis of acupuncture for OSAs, evaluating the efficacy and safety of acupuncture according to the severity of the disease in 2020. The subjects in this review^[25]^ were patients with OSAs, without additional restrictions, and there may be heterogeneity due to different underlying diseases. As far as we know, stroke patients often have SAs. Active intervention of SAs is one of the important measures to improve the prognosis and quality of life for them. At the same time, targeting this population as the subjects will also more accurately evaluate the efficacy and safety of acupuncture. To provide evidence-based medical evidence, we conducted a systematic review and meta-analysis of these randomized controlled trials (RCTs).

## 2. Materials and methods

This systematic review and meta-analysis was performed in accordance with the preferred reporting items for systematic reviews and meta-analyses schema and was registered with INPLASY (registration number: INPLASY202250113).

### 2.1. Criteria for inclusion

#### 2.1.1. Types of studies

The language of published RCT articles was limited to English and Chinese.

#### 2.1.2. Study population

Patients should be diagnosed with stroke and SAs. There were no restrictions on variables such as the type of stroke or SAs, age, sex, race, education, and socioeconomic status.

#### 2.1.3. Measures of intervention

Intervention measures in the treatment group included acupuncture alone or in combination with other therapies. There were no restrictions on variables including acupuncture point selection principles, techniques, needle specifications, and electroacupuncture (EA). The control group received the usual treatment for stroke and other diseases, but did not include acupuncture.

#### 2.1.4. Outcomes

The primary outcome assessed was the total effective rate and AHI. Secondary outcome measures included the minimal oxygen saturation (SaO_2_min), longest apnea time, Epworth sleepiness scale (ESS) score, and adverse reactions. The safety index was defined as the occurrence of adverse events.

### 2.2. Criteria for exclusion

Studies with duplicate data, without full text, or in which the required data could not be obtained from the original literature were excluded. Patients were also excluded if the intervention in the treatment group was transcutaneous electrical acupoint stimulation, dry needle therapy, or sham needle acupuncture.

### 2.3. Strategies for literature retrieval

The following 8 databases were searched: PubMed, Cochrane Library (CENTRAL), Embase, Web of Science, China National Knowledge Infrastructure, Chongqing VIP Information, WanFang Data, and China Biomedical Literature Database. The search terms included stroke, cerebrovascular accident, apoplexy, sleep apnea syndromes, sleep-disordered breathing, acupuncture, pharmacopuncture, and acupotomy. The search strategies are included in the supplementary material. (See Supplemental Digital Content, http://links.lww.com/MD/I657, explaining the search strategy of this study). The search time limit was from the establishment of each database to May 4, 2022. Subject headings, free words, and keywords were used for the retrieval. In addition, relevant literature was supplemented by consulting other resources.

### 2.4. Literature screening and data extraction

Two researchers (Gao and Wang) independently screened the literature, extracted the data, and cross-checked it. In case of disagreement, a third investigator (Kan) was consulted to help make a consensus. Agreement between authors was determined by using Cohen Kappa value. The content of data extraction included essential characteristics of the studies (e.g., authors and publication time), crucial aspects of the included patients (e.g., age, sex, number of cases, and diagnostic criteria), and the key elements of the literature quality evaluation, interventions, treatment courses, and outcomes. For articles in which the full text was not available in the search, the authors were contacted by email or a literature search platform was sought. If there was no response, the study was excluded.

### 2.5. Assessment of risk of bias

Our study assessed the risk of bias in the included studies using the Cochrane risk of bias tool,^[26]^ which is consisted of random sequence generation, allocation concealment, blinding of participants and personnel, blinding of outcome assessment, selective reporting, incomplete outcome data, and other risks of bias, in which the “other risks of bias” were included as follows: whether there were no differences in baseline characteristics and whether there were some relationships of interest. For each RCT included, the aforementioned 7 items were judged as high risk of bias, low risk of bias, or uncertain risk of bias. This process was completed by 2 investigators (Gao and Kan) independently, and in case of inconsistency, a third investigator (Fang) assisted in the judgment.

### 2.6. Statistical processing

RevMan 5.4 software (The Cochrane Collaboration, 2020) was used to perform the meta-analysis. Dichotomous data are presented as the effective rate, relative risk, and 95% confidence interval (CI). Continuous variables are expressed as the mean difference (MD) or standard mean difference with 95% CI. Results were considered significant at *P* < .05. The *Q* test was used to test the heterogeneity of each study; when *P* > .05 and *I*^2^ < 50%, a fixed-effects model was used with good statistical homogeneity. However, when *P* < .05 or *I*^2^ > 50%, considerable heterogeneity existed between the studies; therefore, we used a random-effects model. If heterogeneity was identified through sensitivity or subgroup analyses, fixed-effect models were also used after the exclusion of relevant studies.

### 2.7. Analysis of publication bias

A funnel plot was used to observe publication bias when > 10 related studies corresponded to the outcome index.

## 3. Results and discussion

### 3.1. Search results

**Figure 1.**
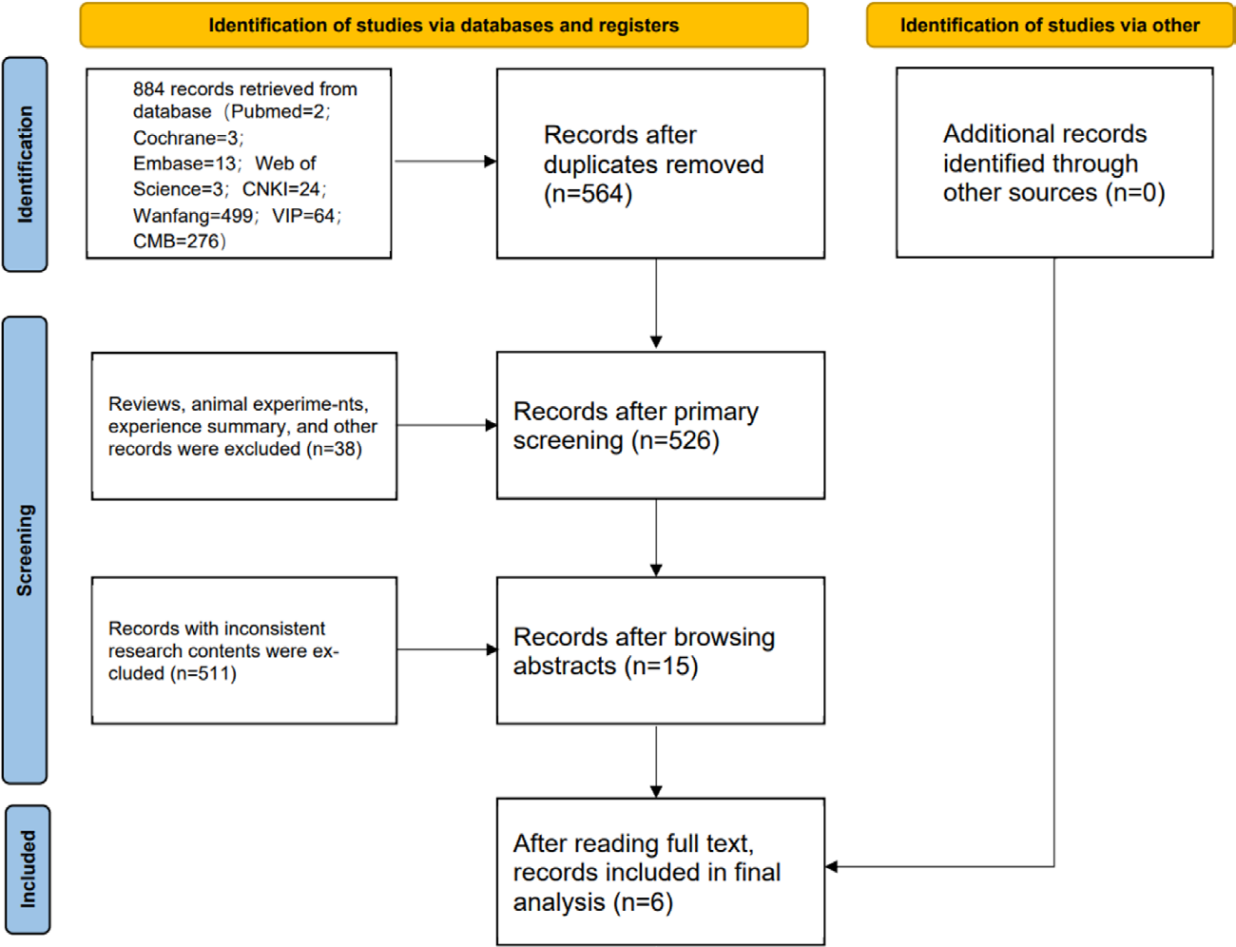
PRISMA flow diagram of the study process. PRISMA = preferred reporting items for systematic review and meta-analysis.

In total, 884 articles were retrieved from the databases. After checking for duplicate, 564 articles were obtained. After excluding reviews, systematic reviews, animal experiments, experience summaries, and guidelines, a total of 38 articles were removed. Fifteen articles were selected after reading the titles and abstracts. Eventually, after reading the full text, 6 records were included.^[27–32]^ Table 1 consists the number of articles extracted from each database. There were 1 English article^[27]^ and 5 Chinese articles^[28–32]^ in the systematic review. The preferred reporting items for systematic reviews and meta-analyses flow chart shows the selection process (Fig. 1).

**Table 1:** The number of articles from each database.

Database	Number
PubMed	1
China National Knowledge Infrastructure	4
WanFang	1

### 3.2. Basic information of all the studies

Six studies included 513 participants, with 256 in the experimental group and 257 in the control group. One study^[28]^ did not specify the stroke type. There were 376 cases of ischemic stroke and 53 cases of hemorrhagic stroke in the remaining studies. All the studies dealt with OSAs. Four studies used general acupuncture,^[28,30–32]^ and 1 combined moxibustion.^[30]^ EA was used in 2 studies,^[27,29]^ one of which combined oral muscle biofeedback system training.^[29]^ Regarding acupuncture methods, only 1 study described manipulation and reinforcing or reducing operations using twirling and reinforcing strategies.^[27]^ Two studies used a combination of the twirling, lifting, and thrusting techniques. However, none of these studies described reinforcing or reducing operations.^[31,32]^ While the gentle reinforcing and reducing technique was performed in 2 studies, the manipulation was unknown.^[28,30]^ One study did not mention this.^[29]^ Between the 2 studies using EA, 1 used an intermittent wave of 120 to 240 times/minutes^[29]^ and the other used a frequency of 120 times/minutes without describing the specific waveform.^[27]^ Lianquan, which was the acupoint with the highest frequency, was used in all 5 articles.^[27,28,30–32]^ In 1 study, the needle retention time was 25 minutes,^[29]^ while in others, it was 30 minutes.^[27,28,30–32]^ The agreement between reviewers reached a kappa value of 0.75. Table 2 presents basic information of the included studies.

**Table 2:** Characteristics of the included studies.

ID	Study	Sample size (cases) experimental group/control group	Gender group (male/female)	Age (yr) experimental group/control group	Diagnostic criteria	Types of stroke ischemic/hemorrhagic	Interventions experimental group/control group	Types of SAs	Acupoints	Operation	Treatment time	Treatment cycle	Outcomes
1	Zhang 2021	44/44	Experimental group (24/20) control group (19/25)	56.80 ± 5.30/5 7.40 ± 6.40	①②	42/46	①②/①	OSAHS	①②	Reinforcing method; Electroacupuncture: the frequency is 120 times /min with unknown	30 min	3 wk	①②④⑤⑦⑧
2	Huang 2018	28/28	Experimental group (19/9) control group (17/11)	63.60 ± 7.00/6 1.30 ± 9.10	②③	49/7	①②③⑤/①③④	OSAHS	②③	Electroacupuncture: the frequency is 120-240 times/min with intermittent wave	25 min	6 wk	②③
3	Wang 2015	63/63	Experimental group (35/28) control group (35/28)	64.00 ± 13.00/63.00 ± 14.00	②④	126/0	①④⑥⑧/①④	OSAHS	①④⑤⑥⑦⑧⑨⑩⑪⑫	Twisting, Lifting and sticking	30 min	30 d	①②③④
4	Lv 2017	40/39	Experimental group (23/17) control group (21/18)	65.20 ± 11.50/64.80 ± 11.40	The article did not describe	79/0	①④⑥⑧/①④	OSAHS	①④⑤⑥⑦⑧⑨⑩⑪⑫	Twisting, Lifting and sticking	30 min	30 d	①②③④
5	Liu 2018	40/40	Experimental group (22/18) control group (21/19)	50.12 ± 5.12/49.89 ± 5.80	②③	80/0	①②④⑦/①④	OSAHS	①④⑤⑬⑭⑮⑯⑰⑱	Gentle reinforcing and reducing technique	30 min	8 wk	①②③⑤⑥⑦⑧
6	Chen 2020	42/42	Experimental group (23/19) control group (22/20)	59.66 ± 4.27/59.58 ± 4.23	The article did not describe	The article did not describe	①②/①	OSAHS	①	Gentle reinforcing and reducing technique	30 min	1 m	①②⑤⑥

Explanation: Diagnostic criteria: ①“Chinese acute ischemic stroke diagnosis and treatment guidelines2014” ② “Guidelines for the diagnosis and treatment of obstructive sleep apnea hypop-nea syndrome (2011 Revision)”③“Diagnostic key points of various cerebrovascular diseases” ④“Chinese acute ischemic stroke diagnosis and treatment guidelines 2010”.

Interventions: ①conventional medical treatment; ②electric acupuncture; ③rehabilitation training; ④continuous positive airway pressure; ⑤oral muscle biofeedback system training; ⑥acupuncture; ⑦moxibustion; ⑧scalp acupuncture.

Outcomes: ①The total effective rate; ②AHI; ③ESS; ④The minimal oxygen saturation (SaO2min ); ⑤Longest apnea time; ⑥The longest duration of apnea; ⑦NSE; ⑧S100β.

Acupoints: ① Lianquan; ②Lianquan side; ③Tiantu side; ④Baihui; ⑤Sishencong; ⑥Tiantu; ⑦Fengchi; ⑧Lieque; ⑨Yinlingquan; ⑩Zhaohai; ⑪Taixi; ⑫Sensory and motor areas of the scalp; ⑬Zusanli; ⑭Taichong; ⑮ Yintang; ⑯ Shenmen; ⑰Sanyinjiao; ⑱Neiguan.

SAs = sleep apnea syndrome.

### 3.3. The quality evaluation results of all studies

**Figure 2.**
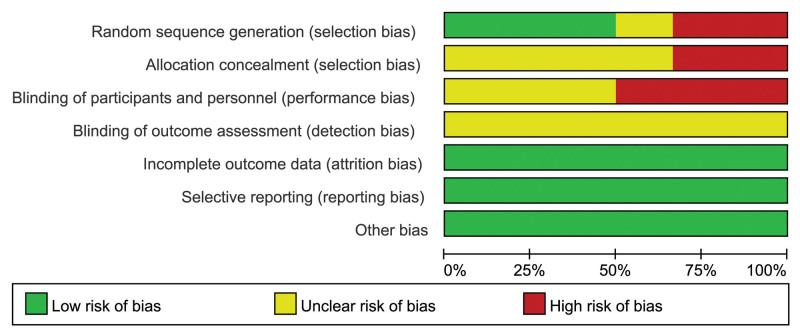
Risk of bias summary.

**Figure 3.**
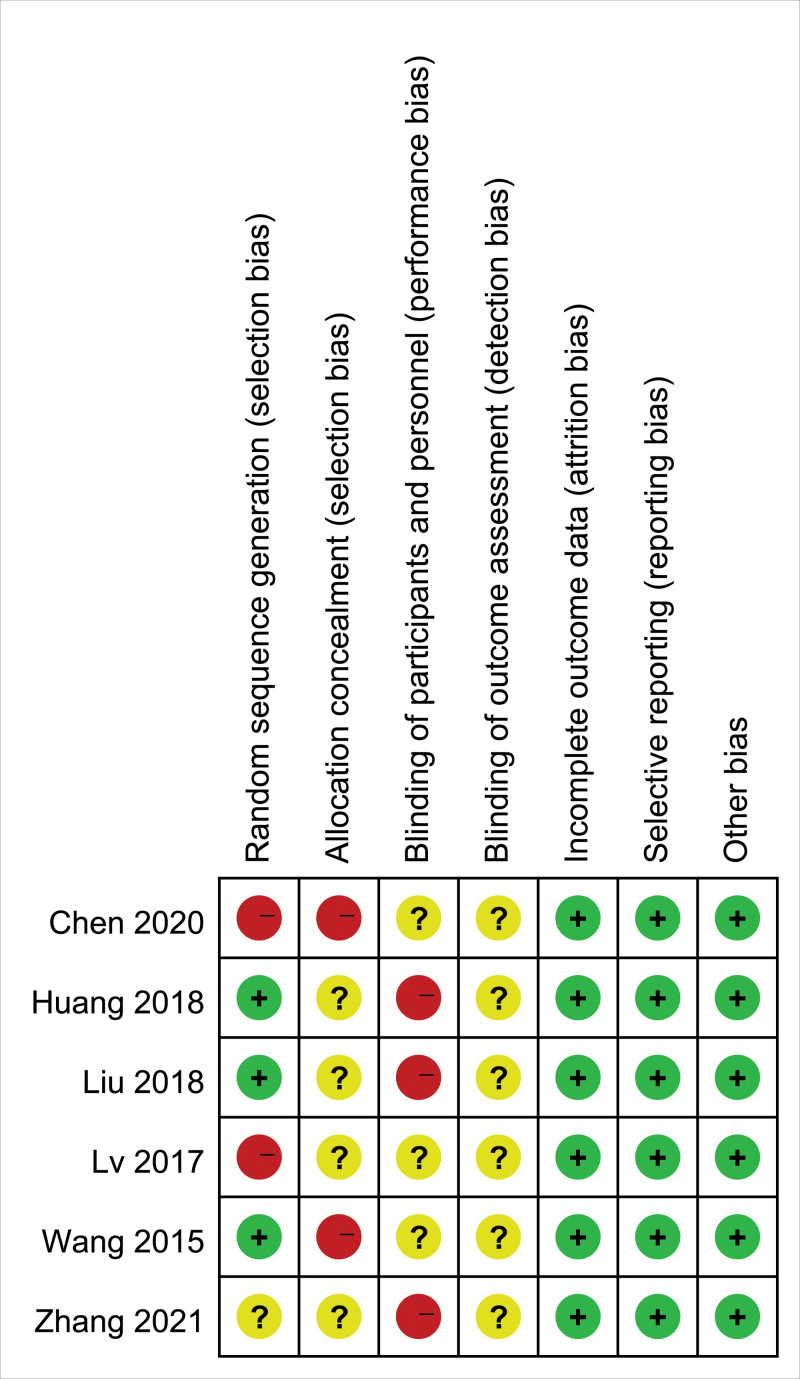
Risk of bias graph.

All included studies were RCTs that mentioned randomization, and 3 used the random number table method^[29,30,32]^; however, 2 studies did not adhere to random principles.^[28,31]^In 1 study, randomization was not clearly described.^[27]^ Two studies failed to implement allocation concealment,^[28,32]^ whereas in the other 4 studies, the situation was not clearly described.^[27,29–31]^ Blinding was not performed in 3 studies^[27,29,30]^ and was not described in detail in the other studies.^[28,31,32]^ None of the studies had case shedding, selective reporting of outcome measures, or other sources of bias, as shown in Figures 2 and 3.

### 3.4. Results of the outcome measures

#### 3.4.1. Total effective rate

**Figure 4.**
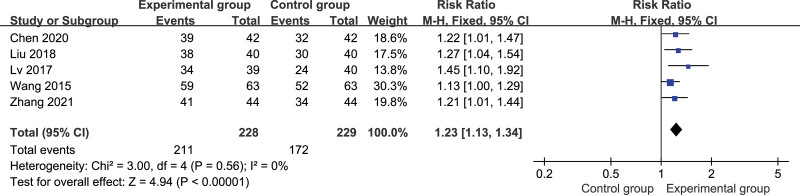
Forest plot of total effective rate in experiment compared with control.

Five studies, including 457 patients, reported the overall response rate.^[27,28,30–32]^ There was no heterogeneity among the results of the studies (*P* = .56, *I*^2^ = 0%); therefore, the fixed- effect model could be used for meta-analysis. The results showed that the total effective rate of the experimental group was significantly higher than that of the control group, and the difference was statistically significant (relative risk = 1.23, 95% CI: 1.13, 1.34, *P* < .00001; Fig. 4). Further sensitivity analysis showed that the results remained unchanged after excluding 1 study, indicating stable results. (See Figure S1–5, Supplemental Digital Content, http://links.lww.com/MD/I658, showing sensitivity analysis of total effective rate.)

#### 3.4.2. AHI

**Figure 5.**
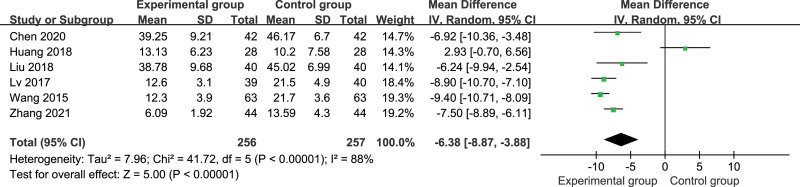
Forest plot of AHI in experiment compared with control. AHI = apnea-hypopnea index.

**Figure 6.**
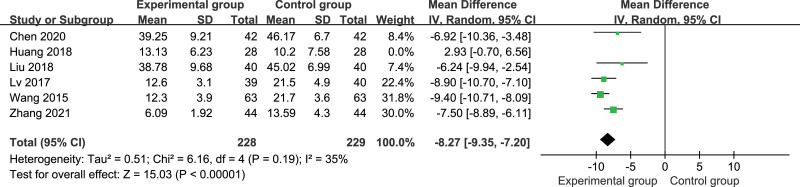
Forest plots of AHI with heterogeneity were removed by sensitivity analysis in the experiment compared with control. AHI = apnea-hypopnea index.

The AHI was reported in all studies,^[27–32]^ including 513 patients. There was heterogeneity among the studies (*P* < .00001, *I*^2^ = 88%); therefore, the random-effects model was used for meta-analysis. The results showed that the AHI was significantly lower in the control group than in the experimental group (MD = −6.38, 95% CI: −8.87, −3.88, *P* < .00001; Fig. 5). A sensitivity analysis was performed by excluding 1 study at a time. (See Figure S6–10, Supplemental Digital Content, http://links.lww.com/MD/I659, showing sensitivity analysis of AHI.) Heterogeneity reduced after Huang study^[29]^ was excluded (*P* = .19, *I*^2^ = 35%). The fixed-effects model was used for meta-analysis again, and the result was consistent with the former: the AHI was significantly lower in the experimental group than in the control group (MD = −8.39, 95% CI: −9.19, −7.59, *P* < .00001; Fig. 6). Considering that the heterogeneity mainly came from Huang study,^[29]^ it may be related to the small sample size of this study or the slightly shorter needle retention time.

#### 3.4.3. ESS score

**Figure 7.**
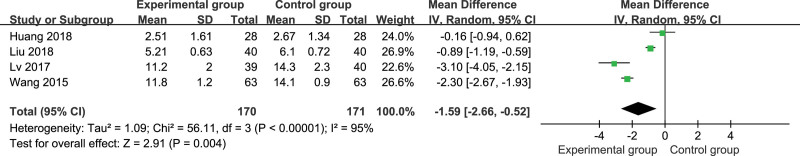
Forest plot of ESS in experiment compared with control. ESS = Epworth sleepiness scale.

The ESS score was reported in 4 studies,^[29–32]^ including 341 patients. Heterogeneity was observed among the studies (*P* < .00001, *I*^2^ = 95%). Therefore, we used a random-effects model for the meta-analysis. The results showed that the ESS score was significantly lower in the experimental group than in the control group (MD = −1.59, 95% CI: −2.66, −0.52, *P* = .004; Fig. 7). Sensitivity analysis was performed by excluding 1 study after another (See Figure S11–14, Supplemental Digital Content, http://links.lww.com/MD/I660, showing sensitivity analysis of ESS score.); however, no source of heterogeneity was found, which may be related to the subjectivity of the ESS score criteria.

#### 3.4.4. SaO_2_min

**Figure 8.**
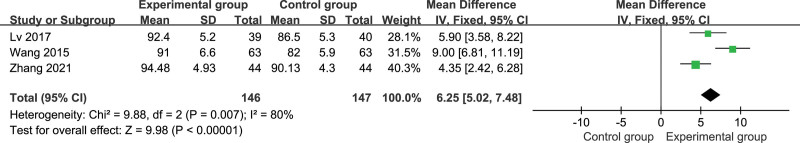
Forest plot of SaO2min in experiment compared with control. SaO2min = minimal oxygen saturation.

**Figure 9.**
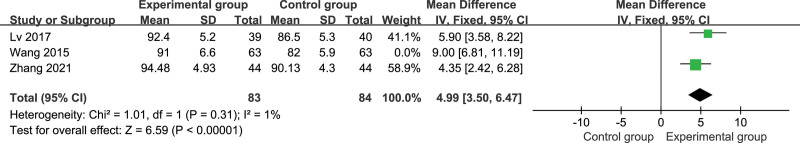
Forest plots of SaO2min with heterogeneity were removed by sensitivity analysis in the experiment compared with control. SaO2min = minimal oxygen saturation.

SaO_2_min was reported in 3 studies,^[27,31,32]^ including 293 patients. Heterogeneity was observed among the studies (*P* < .007, *I*^2^ = 80%). The random-effects model was used for meta-analysis, and the results showed that SaO_2_min was significantly higher in the experimental group than in the control group (MD = 6.39, 95% CI: 3.64, 9.13, *P* < .00001; Fig. 8). A sensitivity analysis was performed by excluding 1 study at a time. (See Figure S15, Supplemental Digital Content, http://links.lww.com/MD/I661,16, http://links.lww.com/MD/I662, showing sensitivity analysis of SaO_2_min.) Heterogeneity was significantly reduced after eliminating Wang study^[32]^ (*P* = .31, *I*^2^ = 1%). Therefore, the fixed-effects model was used for meta-analysis, and the finding was consistent with the former: SaO_2_min was significantly higher in the experimental group than in the control group (MD = 4.99, 95% CI: 3.5, 6.47, *P* < .00001; Fig. 9). The source of the heterogeneity may have been related to the sample themselves.

#### 3.4.5. Longest duration of apnea

**Figure 10.**
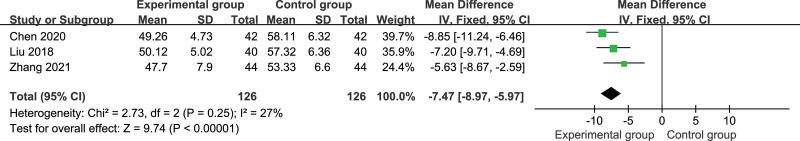
Forest plot of longest duration of apnea in experiment compared with control.

The longest duration of apnea was reported in 3 studies,^[27,28,30]^ including 293 patients. There was no heterogeneity among the results of the studies (*P* = .25, *I*^2^ = 27%); therefore, the fixed-effects model was used for meta-analysis. The longest duration of apnea was significantly shorter in the experimental group than in the control group (MD = −7.47, 95% CI: −8.97, −5.97, *P* < .00001; Fig. 10). Further sensitivity analysis showed that the results remained unchanged after excluding 1 study, indicating stable results. (See Figure S17–19, Supplemental Digital Content, http://links.lww.com/MD/I663, showing sensitivity analysis of longest duration of apnea).

#### 3.4.6. Longest duration of hypoventilation

**Figure 11.**
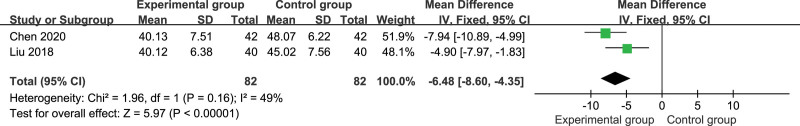
Forest plot of longest duration of hypoventilation in experiment compared with control.

The longest duration of hypoventilation was reported in 2 studies,^[28,30]^ including 164 patients. There was no heterogeneity among the results of the studies (*P* = .16, *I*^2^ = 49%); therefore, the fixed-effects model was used for the meta-analysis. The longest duration of hypoventilation was significantly lower in the experimental group than in the control group (MD = −6.48, 95% CI: −8.60, −4.35, *P* < .00001; Fig. 11).

#### 3.4.7. Neuron-specific enolase

**Figure 12.**
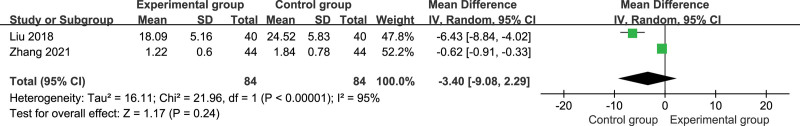
Forest plot of NSE in experiment compared with control. NSE = neuron-specific enolase.

Neuron-specific enolase (NSE) levels were reported in 2 studies,^[27,30]^ including 168 patients. Heterogeneity was found among the studies (*P* < .00001, *I*^2^ = 95%); therefore, the random-effects model was used for meta-analysis. The NSE level of the experimental group was not statistically different from that of the control group (MD = −3.40, 95% CI: −9.08, 2.29, *P* = .24; Fig. 12).

#### 3.4.8. S100β level

**Figure 13.**
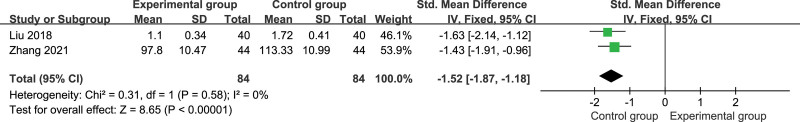
Forest plot of S100β level in experiment compared with control.

The S100β level was reported in 2 studies,^[27,30]^ including 168 patients. There was no heterogeneity among the studies (*P* = .58, *I*^2^ = 0%). Fixed-effects models were used to perform the meta-analysis, which showed that S100β levels in the experimental group were significantly lower than those in the control group (standard mean difference = −1.52, 95% CI: −1.87, −1.18, *P* < .00001; Fig. 13).

#### 3.4.9. Analysis of safety

One study reported treatment safety^[29]^; however, there were no adverse events in the experimental and control groups. Five studies did not describe the safety of treatment,^[27,28,30–32]^ so a meta-analysis could not be performed.

#### 3.4.10. Publication bias

A funnel plot could not be drawn because fewer than 10 studies were included in the analysis. Owing to the small number of studies, publication bias was inevitable in this meta-analysis.

### 3.5. Effectiveness of acupuncture treatment

According to the clinical manifestations of nocturnal snoring, apnea, daytime sleepiness, and lethargy, SAs is classified into “snoring sleep,” “lethargy,” and “phlegm syndrome” in TCM.^[33,34]^ In stroke patients with visceral dysfunction, meridian qi and blood operation are not smooth, and sputum turbidity, blood stasis, and other pathological products block the airway and affect respiratory function. Acupoints such as Lianquan, Lianquan side, Yingxiang, Hegu, Tiantu, Zusanli, Fenglong, and Sanyinjiao are often used for the treatment principles of Xuanfei and Liyan, invigorating spleen and dissipating phlegm. Sometimes acupoints with the function of calming the mind and determining the mind are also used, such as Sishencong, Shenmen, and Baihui.^[35]^ The skin was disinfected with 75% alcohol before needling, and there will be a retention after inserting. Acupuncture can improve the respiratory reflex function by stimulating specific acupoints and mobilizing the movement of qi and blood. This is a therapy that stimulates the nerves and cerebral cortex through acupoints, which is beneficial to restore brain function, thereby enhancing local muscle tone and improving airway collapse.^[36]^ It can also promote local microcirculation and reduce tissue edema, then correct hypoventilation effectively.^[37]^ In addition, some studies have pointed out that acupuncture exerts effects by regulating immune stress response and reducing tissue damage.^[38,39]^ More precise physiological mechanisms need to be explored.

In this meta-analysis, more outcomes were related to SAs. The AHI, SaO_2_min, longest duration of apnea and hypoventilation are essential indicators of sleep apnea detection, and the ESS score is used to evaluate the degree of sleepiness. NSE and S100β levels can reflect the degree of brain tissue damage.^[40,41]^ The results of the meta-analysis showed that acupuncture significantly improved the related indicators of SAs and was superior to the control in reducing the S100β level. (*P* < .05) Nevertheless, the effect of lowering the NSE level was comparable to that of the control (*P* > .05), indicating that acupuncture has an excellent therapeutic effect on SAs. However, its impact on the prognosis of stroke remains unclear.

This result may be related to the acupoint selection scheme. In all the included studies, the frequencies of the Lianquan and Lianquan side were higher. Lianquan is the intersection point of the Ren pulse and Yin Wei pulse, whereas the Lianquan side is 0.5 inches away from it. Both have the effects of opening the orifice and benefiting the pharynx. A previous study reported that the depth of these 2 acupoints is just the base of the tongue with abundant and sensitive nerves and that these acupoints respond strongly after stimulation.^[42]^ These acupoints can significantly improve the function of the pharyngeal dilator muscle and posterior lingual airway.^[43]^ This is a specific point selection for SAs. However, TCM believes that human beings are holistic, and treatment should not only focus on the local but should also be connected with the methods of distal meridian searching, syndrome differentiation, and point selection to realize the coordination of qi operation around the body, which may have a better effect for patients with stroke. Unfortunately, not all of these studies followed this principle, and the therapeutic significance of comprehensive acupoint selection and optimization of the selection plan are worthy of further exploration.

### 3.6. Quality analysis of the RCTs

In this meta-analysis, all included studies mentioned “random” as the grouping method. However, half of the studies described the method of random sequences improperly or vaguely, which increased selectivity bias in the analysis. Allocation concealment prevents allocation outcomes from being known to researchers in advance and exaggerates the treatment effects. According to the literature, 2 studies failed to implement the allocation hiding principle, and 4 did not describe this aspect. Blinding can ensure the authenticity of the results; however, it is difficult to achieve blinding in RCTs due to the characteristics of acupuncture. The results of all studies were complete, with no case shedding, no selective reporting, no difference in baseline between groups before the trial, no benefit relationship with other enterprise projects, and no other bias.

### 3.7. Study limitations

The limitations of this study are as follows; Fewer than 10 studies were finally included, so publication bias was inevitable. Additionally, the sample size of each study was small. The lack of large-scale clinical randomized controlled trials may have affected the objectivity and accuracy of the systematic review; All studies focused on OSAs, and the efficacy of acupuncture for stroke with CSAs remains unclear. Most of the studies failed to describe the severity of the disease accurately, so we could not compare the differences in efficacy laterally; The acupoint selection scheme, needle manipulation, and reinforcing or reducing operations affect the efficacy of acupuncture and moxibustion. Owing to the lack of a detailed description of the original study, we failed to conduct a more in-depth analysis based on these aspects; There are few outcome indicators related to stroke; hence, it is difficult to evaluate the effect of acupuncture on the prognosis of patients with stroke; Most articles do not describe adverse reactions; therefore, it is difficult to analyze the safety of acupuncture in stroke patients with SAs; Long-term follow-up records were lacking in most studies, and long-term effects must be confirmed. These aspects will be explored in the future.

## 4. Conclusions

In this systematic review and meta-analysis, acupuncture effectively improved the total effective rate, apnea-hypopnea index, Epworth Sleepiness Scale score, minimal oxygen saturation, longest duration of apnea, longest duration of apnea, and S100β levels. It was cautiously considered as a treatment for SAs in patients with stroke. Acupuncture can improve clinical symptoms and related laboratory indicators; is cheap, fast, and well tolerated by patients; and is worthy of clinical application. Despite this study’s limitations, it provides directions and a basis for future research. Owing to the shortcomings of the included RCTs, trials with larger sample sizes and more rigorous method designs are needed in the future to explore the efficacy and safety of acupuncture for stroke complicated with SAs. For example, the interventions should be improved in terms of SAs classification, disease severity, acupoint selection scheme, and operation technique. Simultaneously, the long-term effects of acupuncture and the improvement in the prognosis of stroke also need to be confirmed by more in-depth studies.

## Author contributions

**Conceptualization:** Ning Wang, Wenli Yan, Mengqi Yang, Yongmei song.

**Data curation:** Huanyu Gao, Zunqi Kan, Yuqing Fang, Ning Wang, Wenli Yan.

**Formal analysis:** Yuqing Fang, Ning Wang, Wenli Yan.

**Funding acquisition:** Yongmei Song.

**Investigation:** Huanyu Gao, Ning Wang, Yongmei Song.

**Methodology:** Huanyu Gao, Mengqi Yang, Yongmei Song.

**Project administration:** Yongmei Song.

**Resources:** Huanyu Gao, Zunqi Kan.

**Software:** Huanyu Gao, Zunqi Kan, Mengqi Yang.

**Supervision:** Zunqi Kan, Yuqing Fang, Ning Wang, Wenli Yan, Mengqi Yang, Yongmei Song.

**Validation:** Zunqi Kan, Yuqing Fang, Ning Wang.

**Visualization:** Huanyu Gao, Yuqing Fang.

**Writing – original draft:** Huanyu Gao, Zunqi Kan.

**Writing – review & editing:** Huanyu Gao, Zunqi Kan, Yongmei Song.

## Supplementary Material














