# Supplementary material for: Efficacy and safety of acupuncture in the treatment of stroke complicated with sleep apnea syndrome: A systematic review and meta-analysis of randomized controlled trials
Source: Medicine (Baltimore). 2023 Apr 14;102(15):e33241. doi: 10.1097/MD.0000000000033241 (PMC10101308; doi:10.1097/MD.0000000000033241)
Supplement: Supplementary file 1 [file medi-102-e33241-s001.pdf]

Table S1. The search strategy for PubMed	
**Number**	**Search terms**
#1	"Cerebral Hemorrhage"[Mesh]
#2	"Cerebral Hemorrhage"[Title/Abstract]
#3	((((((((((((((((((((((Hemorrhage, Cerebrum[Title/Abstract]) OR (Cerebrum Hemorrhage[Title/Abstract])) OR (Cerebrum Hemorrhages[Title/Abstract])) OR (Hemorrhages, Cerebrum[Title/Abstract])) OR (Cerebral Parenchymal Hemorrhage[Title/Abstract])) OR (Cerebral Parenchymal Hemorrhages[Title/Abstract])) OR (Hemorrhage, Cerebral Parenchymal[Title/Abstract])) OR (Hemorrhages, Cerebral Parenchymal[Title/Abstract])) OR (Parenchymal Hemorrhage, Cerebral[Title/Abstract])) OR (Parenchymal Hemorrhages, Cerebral[Title/Abstract])) OR (Intracerebral Hemorrhage[Title/Abstract])) OR (Hemorrhage, Intracerebral[Title/Abstract])) OR (Hemorrhages, Intracerebral[Title/Abstract])) OR (Intracerebral Hemorrhages[Title/Abstract])) OR (Hemorrhage, Cerebral[Title/Abstract])) OR (Cerebral Hemorrhages[Title/Abstract])) OR (Hemorrhages, Cerebral[Title/Abstract])) OR (Brain Hemorrhage, Cerebral[Title/Abstract])) OR (Brain Hemorrhages, Cerebral[Title/Abstract])) OR (Cerebral Brain Hemorrhage[Title/Abstract])) OR (Cerebral Brain Hemorrhages[Title/Abstract])) OR (Hemorrhage, Cerebral Brain[Title/Abstract])) OR (Hemorrhages, Cerebral Brain[Title/Abstract])
#4	“Cerebral infarction”[MeSH Terms]

#5	Cerebral infarction[Title/Abstract]
#6	((((((((((((((((((((((((Cerebral Infarctions[Title/Abstract]) OR (Infarctions, Cerebral[Title/Abstract])) OR (Infarction, Cerebral[Title/Abstract])) OR (Cerebral Infarct[Title/Abstract])) OR (Cerebral Infarcts[Title/Abstract])) OR (Infarct, Cerebral[Title/Abstract])) OR (Infarcts, Cerebral[Title/Abstract])) OR (Cerebral Infarction, Left Hemisphere[Title/Abstract])) OR (Left Hemisphere, Infarction, Cerebral[Title/Abstract])) OR (Infarction, Left Hemisphere, Cerebral[Title/Abstract])) OR (Left Hemisphere, Cerebral Infarction[Title/Abstract])) OR (Cerebral, Left Hemisphere, Infarction[Title/Abstract])) OR (Infarction, Cerebral, Left Hemisphere[Title/Abstract])) OR (Subcortical Infarction[Title/Abstract])) OR (Infarction, Subcortical[Title/Abstract])) OR (Infarctions, Subcortical[Title/Abstract])) OR (Subcortical Infarctions[Title/Abstract])) OR (Posterior Choroidal Artery Infarction[Title/Abstract])) OR (Anterior Choroidal Artery Infarction[Title/Abstract])) OR (Cerebral Infarction, Right Hemisphere[Title/Abstract])) OR (Right Hemisphere, Cerebral Infarction[Title/Abstract])) OR (Infarction, Right Hemisphere, Cerebral[Title/Abstract])) OR (Right Hemisphere, Infarction, Cerebral[Title/Abstract])) OR (Cerebral, Right Hemisphere, Infarction[Title/Abstract])) OR (Infarction, Cerebral, Right Hemisphere[Title/Abstract])
#7	“stroke”[MeSH Terms]
#8	stroke[Title/Abstract]
#9	(((((((((((((((((((((((((((Strokes[Title/Abstract]) OR (Cerebrovascular Accident[Title/Abstract])) OR (Cerebrovascular

	Accidents[Title/Abstract])) OR (CVA (Cerebrovascular Accident[Title/Abstract]))) OR (CVAs (Cerebrovascular Accident[Title/Abstract]))) OR (Cerebrovascular Apoplexy[Title/Abstract])) OR (Apoplexy, Cerebrovascular[Title/Abstract])) OR (Vascular Accident, Brain[Title/Abstract])) OR (Brain Vascular Accident[Title/Abstract])) OR (Brain Vascular Accidents[Title/Abstract])) OR (Vascular Accidents, Brain[Title/Abstract])) OR (Cerebrovascular Stroke[Title/Abstract])) OR (Cerebrovascular Strokes[Title/Abstract])) OR (Stroke, Cerebrovascular[Title/Abstract])) OR (Strokes, Cerebrovascular[Title/Abstract])) OR (Apoplexy[Title/Abstract])) OR (Cerebral Stroke[Title/Abstract])) OR (Cerebral Strokes[Title/Abstract])) OR (Stroke, Cerebral[Title/Abstract])) OR (Strokes, Cerebral[Title/Abstract])) OR (Stroke, Acute[Title/Abstract])) OR (Acute Stroke[Title/Abstract])) OR (Acute Strokes[Title/Abstract])) OR (Strokes, Acute[Title/Abstract])) OR (Cerebrovascular Accident, Acute[Title/Abstract])) OR (Acute Cerebrovascular Accident[Title/Abstract])) OR (Acute Cerebrovascular Accidents[Title/Abstract])) OR (Cerebrovascular Accidents, Acute[Title/Abstract])
#10	#1 OR #2 OR #3 OR #4 OR #5 OR #6 OR #7 OR #8 OR #9
#11	“Sleep Apnea Syndromes"[Mesh]
#12	Sleep Apnea Syndromes[Title/Abstract]
#13	(((((((((((((((((Apnea Syndrome, Sleep[Title/Abstract]) OR (Apnea Syndromes, Sleep[Title/Abstract])) OR (Sleep Apnea Syndrome[Title/Abstract])) OR (Sleep Hypopnea[Title/Abstract]))

	OR (Hypopnea, Sleep[Title/Abstract])) OR (Hypopneas, Sleep[Title/Abstract])) OR (Sleep Hypopneas[Title/Abstract])) OR (Apnea, Sleep[Title/Abstract])) OR (Apneas, Sleep[Title/Abstract])) OR (Sleep Apnea[Title/Abstract])) OR (Sleep Apneas[Title/Abstract])) OR (Sleep Apnea, Mixed Central[Title/Abstract] AND Obstructive[Title/Abstract])) OR (Mixed Central[Title/Abstract] AND Obstructive Sleep Apnea[Title/Abstract])) OR (Sleep Apnea, Mixed[Title/Abstract])) OR (Mixed Sleep Apnea[Title/Abstract])) OR (Mixed Sleep Apneas[Title/Abstract])) OR (Sleep Apneas, Mixed[Title/Abstract])) OR (Hypersomnia with Periodic Respiration[Title/Abstract])) OR (Sleep-Disordered Breathing[Title/Abstract])) OR (Breathing, Sleep-Disordered[Title/Abstract])) OR (Sleep Disordered Breathing[Title/Abstract])
#14	#11 OR #12 OR #13
#15	"Acupuncture"[Mesh]
#16	Acupuncture[Title/Abstract]
#17	Pharmacopuncture[Title/Abstract]
#18	"Acupuncture Therapy"[Mesh]
#19	Acupuncture Therapy[Title/Abstract]
#20	((((((((Acupuncture Treatment[Title/Abstract]) OR (Acupuncture Treatments[Title/Abstract])) OR (Treatment, Acupuncture[Title/Abstract])) OR (Therapy, Acupuncture[Title/Abstract])) OR (Pharmacoacupuncture Treatment[Title/Abstract])) OR (Treatment, Pharmacoacupuncture[Title/Abstract])) OR (Pharmacoacupuncture Therapy[Title/Abstract])) OR (Therapy, Pharmacoacupuncture[Title/Abstract])) OR

	(Acupotomy[Title/Abstract])) OR (Acupotomies[Title/Abstract])
#21	#15 OR #16 OR #17 OR #18 OR #19 OR #20
#24	#10 AND #14 AND #21
**The search strategy for Embase**	
**Number**	**Search terms**
#1	'brain hemorrhage'/exp
#2	'cerebral hemorrhage':ab,it OR 'hemorrhage, cerebrum':ab,it OR 'cerebrum hemorrhage':ab,it OR 'cerebrum hemorrhages':ab,it OR 'hemorrhages, cerebrum':ab,it OR 'cerebral parenchymal hemorrhage':ab,it OR 'cerebral parenchymal hemorrhages':ab,it OR 'hemorrhage, cerebral parenchymal':ab,it OR 'hemorrhages, cerebral parenchymal':ab,it OR 'parenchymal hemorrhage, cerebral':ab,it OR 'parenchymal hemorrhages, cerebral':ab,it OR 'intracerebral hemorrhage':ab,it OR 'hemorrhage, intracerebral':ab,it OR 'hemorrhages, intracerebral':ab,it OR 'intracerebral hemorrhages':ab,it OR 'hemorrhage, cerebral':ab,it OR 'cerebral hemorrhages':ab,it OR 'hemorrhages, cerebral':ab,it OR 'brain hemorrhage, cerebral':ab,it OR 'brain hemorrhages, cerebral':ab,it OR 'cerebral brain hemorrhage':ab,it OR 'cerebral brain hemorrhages':ab,it OR 'hemorrhage, cerebral brain':ab,it OR 'hemorrhages, cerebral brain':ab,it
#3	'brain infarction'/exp
#4	'cerebral infarction':ab,ti OR 'cerebral infarctions':ab,ti OR 'infarctions, cerebral':ab,ti OR 'infarction, cerebral':ab,ti OR 'cerebral infarct':ab,ti OR 'cerebral infarcts':ab,ti OR 'infarct, cerebral':ab,ti OR 'infarcts, cerebral':ab,ti OR 'cerebral infarction, left hemisphere':ab,ti OR 'left hemisphere, infarction, cerebral':ab,ti OR 'infarction, left hemisphere, cerebral':ab,ti OR 'left hemisphere,

	cerebral infarction':ab,ti OR 'cerebral, left hemisphere, infarction':ab,ti OR 'infarction, cerebral, left hemisphere':ab,ti OR 'subcortical infarction':ab,ti OR 'infarction, subcortical':ab,ti OR 'infarctions, subcortical':ab,ti OR 'subcortical infarctions':ab,ti OR 'posterior choroidal artery infarction':ab,ti OR 'anterior choroidal artery infarction':ab,ti OR 'cerebral infarction, right hemisphere':ab,ti OR 'right hemisphere, cerebral infarction':ab,ti OR 'infarction, right hemisphere, cerebral':ab,ti OR 'right hemisphere, infarction, cerebral':ab,ti OR 'cerebral, right hemisphere, infarction':ab,ti OR 'infarction, cerebral, right hemisphere':ab,ti
#5	'cerebrovascular accident'/exp
#6	'stroke':ab,ti OR 'strokes':ab,ti OR 'cerebrovascular accident':ab,ti OR 'cerebrovascular accidents':ab,ti OR 'cva (cerebrovascular accident)':ab,ti OR 'cvas (cerebrovascular accident)':ab,ti OR 'cerebrovascular apoplexy':ab,ti OR 'apoplexy, cerebrovascular':ab,ti OR 'vascular accident, brain':ab,ti OR 'brain vascular accident':ab,ti OR 'brain vascular accidents':ab,ti OR 'vascular accidents, brain':ab,ti OR 'cerebrovascular stroke':ab,ti OR 'cerebrovascular strokes':ab,ti OR 'stroke, cerebrovascular':ab,ti OR 'strokes, cerebrovascular':ab,ti OR 'apoplexy':ab,ti OR 'cerebral stroke':ab,ti OR 'cerebral strokes':ab,ti OR 'stroke, cerebral':ab,ti OR 'strokes, cerebral':ab,ti OR 'stroke, acute':ab,ti OR 'acute stroke':ab,ti OR 'acute strokes':ab,ti OR 'strokes, acute':ab,ti OR 'cerebrovascular accident, acute':ab,ti OR 'acute cerebrovascular accident':ab,ti OR 'acute cerebrovascular accidents':ab,ti OR 'cerebrovascular accidents, acute':ab,ti
#7	'sleep disordered breathing'/exp

#8	'Sleep Apnea Syndromes':ab,ti OR 'Apnea Syndrome, Sleep':ab,ti OR 'Apnea Syndromes, Sleep':ab,ti OR 'Sleep Apnea Syndrome':ab,ti OR 'Sleep Hypopnea':ab,ti OR 'Hypopnea, Sleep':ab,ti OR 'Hypopneas, Sleep':ab,ti OR 'Sleep Hypopneas':ab,ti OR 'Apnea, Sleep':ab,ti OR 'Apneas, Sleep':ab,ti OR 'Sleep Apnea':ab,ti OR 'Sleep Apneas':ab,ti OR 'Sleep Apnea, Mixed Central and Obstructive':ab,ti OR 'Mixed Central and Obstructive Sleep Apnea':ab,ti OR 'Sleep Apnea, Mixed':ab,ti OR 'Mixed Sleep Apnea':ab,ti OR 'Mixed Sleep Apneas':ab,ti OR 'Sleep Apneas, Mixed':ab,ti OR 'Hypersomnia with Periodic Respiration':ab,ti OR 'Sleep-Disordered Breathing':ab,ti OR 'Breathing, Sleep-Disordered':ab,ti OR 'Sleep Disordered Breathing':ab,ti
#9	'acupuncture'/exp
#10	'acupuncture':ab,ti OR 'Pharmacopuncture':ab,ti OR 'Acupuncture Treatment':ab,ti OR 'Acupuncture Treatments':ab,ti OR 'Treatment, Acupuncture':ab,ti OR 'Therapy, Acupuncture':ab,ti OR 'Pharmacoacupuncture Treatment':ab,ti OR 'Treatment, Pharmacoacupuncture':ab,ti OR 'Pharmacoacupuncture Therapy':ab,ti OR 'Therapy, Pharmacoacupuncture':ab,ti OR 'Acupotomy':ab,ti OR 'Acupotomies':ab,ti
#11	#1 OR #2 OR #3 OR #4 OR#5 OR #6
#12	#7 OR #8
#13	#9 OR #10
#14	#11 AND #12 AND #13
**The search strategy for Cochrane Library**	
**Number**	**Search terms**
#1	MeSH descriptor: [Cerebral Hemorrhage] explode all trees
#2	(Cerebral Hemorrhage):ti,ab,kw

#3	(Hemorrhage, Cerebrum):ti,ab,kw OR (Cerebrum Hemorrhage):ti,ab,kw OR (Cerebrum Hemorrhages):ti,ab,kw OR (Hemorrhages, Cerebrum):ti,ab,kw OR (Cerebral Parenchymal Hemorrhage):ti,ab,kw OR (Cerebral Parenchymal Hemorrhages):ti,ab,kw OR (Hemorrhage, Cerebral Parenchymal):ti,ab,kw OR (Hemorrhages, Cerebral Parenchymal):ti,ab,kw OR (Parenchymal Hemorrhage, Cerebral):ti,ab,kw OR (Parenchymal Hemorrhages, Cerebral):ti,ab,kw OR (Intracerebral Hemorrhage):ti,ab,kw OR (Hemorrhage, Intracerebral):ti,ab,kw OR (Hemorrhages, Intracerebral):ti,ab,kw OR (Intracerebral Hemorrhages):ti,ab,kw OR (Hemorrhage, Cerebral):ti,ab,kw OR (Cerebral Hemorrhages):ti,ab,kw OR (Hemorrhages, Cerebral):ti,ab,kw OR (Brain Hemorrhage, Cerebral):ti,ab,kw OR (Brain Hemorrhages, Cerebral):ti,ab,kw OR (Cerebral Brain Hemorrhage):ti,ab,kw OR (Cerebral Brain Hemorrhages):ti,ab,kw OR (Hemorrhage, Cerebral Brain):ti,ab,kw OR (Hemorrhages, Cerebral Brain):ti,ab,kw
#4	MeSH descriptor: [Cerebral Infarction] explode all trees
#5	(Cerebral Infarction):ti,ab,kw
#6	(Cerebral Infarctions):ti,ab,kw OR (Infarctions, Cerebral):ti,ab,kw OR (Infarction, Cerebral):ti,ab,kw OR (Cerebral Infarct):ti,ab,kw OR (Cerebral Infarcts):ti,ab,kw OR (Infarct, Cerebral):ti,ab,kw OR (Infarcts, Cerebral):ti,ab,kw OR (Cerebral Infarction, Left Hemisphere):ti,ab,kw OR (Left Hemisphere, Infarction, Cerebral):ti,ab,kw OR (Infarction, Left Hemisphere, Cerebral):ti,ab,kw OR (Left Hemisphere, Cerebral Infarction):ti,ab,kw OR (Cerebral, Left Hemisphere, Infarction):ti,ab,kw OR (Infarction, Cerebral, Left Hemisphere):ti,ab,kw OR (Subcortical Infarction):ti,ab,kw OR

	(Infarction, Subcortical):ti,ab,kw OR (nfarctions, Subcortical):ti,ab,kw OR (Subcortical Infarctions):ti,ab,kw OR (Posterior Choroidal Artery Infarction):ti,ab,kw OR (Anterior Choroidal Artery Infarction):ti,ab,kw OR (Cerebral Infarction, Right Hemisphere):ti,ab,kw OR (Right Hemisphere, Cerebral Infarction):ti,ab,kw OR (Infarction, Right Hemisphere, Cerebral):ti,ab,kw OR (Right Hemisphere, Infarction, Cerebral):ti,ab,kw OR (Cerebral, Right Hemisphere, Infarction):ti,ab,kw OR (Infarction, Cerebral, Right Hemisphere):ti,ab,kw
#7	MeSH descriptor: [Stroke] explode all trees
#8	(Stroke):ti,ab,kw
#9	(Strokes):ti,ab,kw OR (Cerebrovascular Accident):ti,ab,kw OR (Cerebrovascular Accidents):ti,ab,kw OR (CVA (Cerebrovascular Accident)):ti,ab,kw OR (CVAs (Cerebrovascular Accident)):ti,ab,kw OR (Cerebrovascular Apoplexy):ti,ab,kw OR (Apoplexy, Cerebrovascular):ti,ab,kw OR (Vascular Accident, Brain):ti,ab,kw OR (Brain Vascular Accident):ti,ab,kw OR (Brain Vascular Accidents):ti,ab,kw OR (Vascular Accidents, Brain):ti,ab,kw OR (Cerebrovascular Stroke):ti,ab,kw OR (Cerebrovascular Strokes):ti,ab,kw OR (Stroke, Cerebrovascular):ti,ab,kw OR (Strokes, Cerebrovascular):ti,ab,kw OR (Apoplexy):ti,ab,kw OR (Cerebral Stroke):ti,ab,kw OR (Cerebral Strokes):ti,ab,kw OR (Stroke, Cerebral):ti,ab,kw OR (Strokes, Cerebral):ti,ab,kw OR (Stroke, Acute):ti,ab,kw OR (Acute Stroke):ti,ab,kw OR (Acute Strokes):ti,ab,kw OR (Strokes, Acute):ti,ab,kw OR (Cerebrovascular Accident, Acute):ti,ab,kw OR (Acute Cerebrovascular Accident):ti,ab,kw OR (Acute Cerebrovascular Accidents):ti,ab,kw OR (Cerebrovascular

	Accidents, Acute):ti,ab,kw
#10	#1 OR #2 OR #3 OR #4 OR #5 OR #6 OR #7 OR #8 OR #9
#11	MeSH descriptor: [Sleep Apnea Syndromes] explode all trees
#12	(Sleep Apnea Syndromes):ti,ab,kw
#13	(Apnea Syndrome, Sleep):ti,ab,kw OR (Apnea Syndromes, Sleep):ti,ab,kw OR (Sleep Apnea Syndrome):ti,ab,kw OR (Sleep Hypopnea):ti,ab,kw OR (Hypopnea, Sleep):ti,ab,kw OR (Hypopneas, Sleep):ti,ab,kw OR (Sleep Hypopneas):ti,ab,kw OR (Apnea, Sleep):ti,ab,kw OR (Apneas, Sleep):ti,ab,kw OR (Sleep Apnea):ti,ab,kw OR (Sleep Apneas):ti,ab,kw OR (Sleep Apnea, Mixed Central and Obstructive):ti,ab,kw OR (Mixed Central and Obstructive Sleep Apnea):ti,ab,kw OR (Sleep Apnea, Mixed):ti,ab,kw OR (Mixed Sleep Apnea):ti,ab,kw OR (Mixed Sleep Apneas):ti,ab,kw OR (Sleep Apneas, Mixed):ti,ab,kw OR (Hypersomnia with Periodic Respiration):ti,ab,kw OR (Sleep-Disordered Breathing):ti,ab,kw OR (Breathing, Sleep-Disordered):ti,ab,kw OR (Sleep Disordered Breathing):ti,ab,kw
#14	#11 OR #12 OR #13
#15	#10 AND #14
#16	MeSH descriptor: [Acupuncture] explode all trees
#17	(Acupuncture):ti,ab,kw
#18	(Pharmacopuncture):ti,ab,kw
#19	MeSH descriptor: [Acupuncture Therapy] explode all trees
#20	(Acupuncture Therapy):ti,ab,kw
#21	(Acupuncture Treatment):ti,ab,kw OR (Acupuncture Treatments):ti,ab,kw OR (Treatment, Acupuncture):ti,ab,kw OR (Therapy, Acupuncture):ti,ab,kw OR (Pharmacoacupuncture Treatment):ti,ab,kw OR (Treatment, Pharmacoacupuncture):ti,ab,kw OR (Pharmacoacupuncture

	Therapy):ti,ab,kw OR (Therapy, Pharmacoacupuncture):ti,ab,kw OR (Acupotomy):ti,ab,kw OR (Acupotomies):ti,ab,kw
#22	#16 OR #17 OR #18 OR #19 OR #20 OR #21
#23	#15 AND #22
**The search strategy for Web of Science**	
**Number**	**Search terms**
#1	TS=(Cerebral infarction OR Cerebral Infarctions OR Infarctions, Cerebral OR Infarction, Cerebral OR Cerebral Infarct OR Cerebral Infarcts OR Infarct, Cerebral OR Infarcts, Cerebral OR Cerebral Infarction, Left Hemisphere OR Left Hemisphere, Infarction, Cerebral OR Infarction, Left Hemisphere, Cerebral OR Left Hemisphere, Cerebral Infarction OR Cerebral, Left Hemisphere, Infarction OR Infarction, Cerebral, Left Hemisphere OR Subcortical Infarction OR Infarction, Subcortical OR Infarctions, Subcortical OR Subcortical Infarctions OR Posterior Choroidal Artery Infarction OR Anterior Choroidal Artery Infarction OR Cerebral Infarction, Right Hemisphere OR Right Hemisphere, Cerebral Infarction OR Infarction, Right Hemisphere, Cerebral OR Right Hemisphere, Infarction, Cerebral OR Cerebral, Right Hemisphere, Infarction OR Infarction, Cerebral, Right Hemisphere OR stroke OR Strokes OR Cerebrovascular Accident OR Cerebrovascular Accidents OR CVA (Cerebrovascular Accident) OR CVAs (Cerebrovascular Accident) OR Cerebrovascular Apoplexy OR Apoplexy, Cerebrovascular OR Vascular Accident, Brain OR Brain Vascular Accident OR Brain Vascular Accidents OR Vascular Accidents, Brain OR Cerebrovascular Stroke OR Cerebrovascular Strokes OR Stroke, Cerebrovascular OR Strokes, Cerebrovascular OR Apoplexy OR Cerebral Stroke OR Cerebral Strokes OR Stroke, Cerebral OR Strokes, Cerebral OR Stroke,

	Acute OR Acute Stroke OR Acute Strokes OR Strokes, Acute OR Cerebrovascular Accident, Acute OR Acute Cerebrovascular Accident OR Acute Cerebrovascular Accidents OR Cerebrovascular Accidents, Acute OR Cerebrum Hemorrhage OR Cerebrum Hemorrhages OR Hemorrhages, Cerebrum OR Cerebral Parenchymal Hemorrhage OR Cerebral Parenchymal Hemorrhages OR Hemorrhage, Cerebral Parenchymal OR Hemorrhages, Cerebral Parenchymal OR Parenchymal Hemorrhage, Cerebral OR Parenchymal Hemorrhages, Cerebral OR Intracerebral Hemorrhage OR Hemorrhage, Intracerebral OR Hemorrhages, Intracerebral OR Intracerebral Hemorrhages OR Hemorrhage, Cerebral OR Cerebral Hemorrhages OR Hemorrhages, Cerebral OR Brain Hemorrhage, Cerebral OR Brain Hemorrhages, Cerebral OR Cerebral Brain Hemorrhage OR Cerebral Brain Hemorrhages OR Hemorrhage, Cerebral Brain OR Hemorrhages, Cerebral Brain)
#2	TS=(Sleep Apnea Syndromes OR Apnea Syndrome, Sleep OR Apnea Syndromes, Sleep OR Sleep Apnea Syndrome OR Sleep Hypopnea OR Hypopnea, Sleep OR Hypopneas, Sleep OR Sleep Hypopneas OR Apnea, Sleep OR Apneas, Sleep OR Sleep Apnea OR Sleep Apneas OR Sleep Apnea, Mixed Central and Obstructive OR Mixed Central and Obstructive Sleep Apnea OR Sleep Apnea, Mixed OR Mixed Sleep Apnea OR Mixed Sleep Apneas OR Sleep Apneas, Mixed OR Hypersomnia with Periodic Respiration OR Sleep-Disordered Breathing OR Breathing, Sleep-Disordered OR Sleep Disordered Breathing)
#3	TS=(Acupuncture OR Pharmacopuncture OR Acupuncture Therapy OR Acupuncture Treatment OR Acupuncture Treatments OR Treatment, Acupuncture OR Therapy, Acupuncture OR Pharmacoacupuncture Treatment OR Treatment,

	Pharmacoacupuncture OR Pharmacoacupuncture Therapy OR Therapy, Pharmacoacupuncture OR Acupotomy OR Acupotomies)
#4	#1 AND #2 AND #3
**The search strategy for CNKI**	
(SU = '脑卒中' OR SU = '卒中' OR SU = '缺血性脑卒中' OR SU = '缺血性脑血管病' OR SU = '出血性脑卒中' OR SU = '出血性脑血管病' OR SU = '脑血管意外' OR SU = '脑血管病' OR SU = '缺血性脑中风' OR SU = '出血性脑中风' OR SU = '脑中风' OR SU = '中风' OR SU = '脑梗' OR SU = '脑血栓' OR SU = '脑出血') AND (SU='睡眠呼吸暂停综合征' OR SU = '睡眠呼吸暂停综合症' OR SU = '低通气综合征' OR SU = '睡眠呼吸紊乱' OR SU = '睡眠呼吸暂停' OR SU = '睡眠呼吸障碍' OR SU = '睡眠呼吸不足' OR SU = '混合性睡眠呼吸暂停' OR SU = '阻塞性睡眠呼吸暂停' OR SU = '中枢性睡眠呼吸暂停' OR SU = '睡眠障碍性呼吸' OR SU = '鼾症' OR SU = 'SAHS' OR SU = 'SAS') AND (SU= '针刺' OR SU = '针刺法' OR SU = '针刺治疗' OR SU = '针刺疗法' OR SU = '针刺临床' OR SU = '针灸' OR SU = '针灸法' OR SU = '针灸治疗' OR SU = '针灸疗法' OR SU = '针灸临床' OR SU = '针')	
**The search strategy for WanFang**	
主题:("脑卒中" or "卒中" or "缺血性脑卒中" or "缺血性脑血管病" or "出血性脑卒中" or "出血性脑血管病" or "脑血管意外" or "脑血管病" or "缺血性脑中风" or "出血性脑中风" or "脑中风" or "中风" or "脑梗" or "脑血栓" or "脑出血") and 主题:("睡眠呼吸暂停综合征" or "睡眠呼吸暂停综合症" or "低通气综合征" or "睡眠呼吸紊乱" or "睡眠呼吸暂停" or "睡眠呼吸障碍" or "睡眠呼吸不足" or "混合性睡眠呼吸暂停" or "阻塞性睡眠呼吸暂停" or "中枢性睡眠呼吸暂停" or "睡眠障碍性呼吸" or "鼾症" or "SAHS" or "SAS") and 主题:("针刺" or "针刺法" or "针刺治疗" or "针刺疗法" or "针刺临床" or "针灸" or "针灸法" or "针灸治疗" or "针灸疗法" or "针灸临床" or "针")	
**The search strategy for VIP**	
((M=脑卒中 OR 卒中 OR 缺血性脑卒中 OR 缺血性脑血管病 OR 出血性	

脑卒中 OR 出血性脑血管病 OR 脑血管意外 OR 脑血管病 OR 缺血性脑中风 OR 出血性脑中风 OR 脑中风 OR 中风 OR 脑梗 OR 脑血栓 OR 脑出血) OR (R=脑卒中 OR 卒中 OR 缺血性脑卒中 OR 缺血性脑血管病 OR 出血性脑卒中 OR 出血性脑血管病 OR 脑血管意外 OR 脑血管病 OR 缺血性脑中风 OR 出血性脑中风 OR 脑中风 OR 中风 OR 脑梗 OR 脑血栓 OR 脑出血)) AND ((M=睡眠呼吸暂停综合征 OR 睡眠呼吸暂停综合症 OR 低通气综合征 OR 睡眠呼吸紊乱 OR 睡眠呼吸暂停 OR 睡眠呼吸障碍 OR 睡眠呼吸不足 OR 混合性睡眠呼吸暂停 OR 阻塞性睡眠呼吸暂停 OR 中枢性睡眠呼吸暂停 OR 睡眠障碍性呼吸 OR 鼾症 OR SAHS OR SAS) OR (R=睡眠呼吸暂停综合征 OR 睡眠呼吸暂停综合症 OR 低通气综合征 OR 睡眠呼吸紊乱 OR 睡眠呼吸暂停 OR 睡眠呼吸障碍 OR 睡眠呼吸不足 OR 混合性睡眠呼吸暂停 OR 阻塞性睡眠呼吸暂停 OR 中枢性睡眠呼吸暂停 OR 睡眠障碍性呼吸 OR 鼾症 OR SAHS OR SAS)) AND ((M=针刺 OR 针刺法 OR 针刺治疗 OR 针刺疗法 OR 针刺临床 OR 针灸 OR 针灸法 OR 针灸治疗 OR 针灸疗法 OR 针灸临床 OR 针) OR (R=针刺 OR 针刺法 OR 针刺治疗 OR 针刺疗法 OR 针刺临床 OR 针灸 OR 针灸法 OR 针灸治疗 OR 针灸疗法 OR 针灸临床 OR 针))	
**The search strategy for CBM**	
#1	“脑梗死”［不加权:扩展］
#2	“脑出血”［不加权:扩展］
#3	“卒中”［不加权:扩展］
#4	“脑卒中” [常用字段:智能] OR “卒中” [常用字段:智能] OR “缺血性脑卒中” [常用字段:智能] OR “缺血性脑血管病” [常用字段:智能] OR “出血性脑卒中” [常用字段:智能] OR “出血性脑血管病” [常用字段:智能] OR “脑血管意外” [常用字段:智能] OR “脑血管病” [常用字段:智能] OR “缺血性脑中风” [常用字段:智能] OR “出血性脑中风” [常用字段:智能] OR “脑中风” [常用字段:智能] OR “中风” [常用字段:智能] OR “脑梗” [常用字段:智能] OR “脑血栓” [常用字段:智能] OR “脑出血”[常用字段:智能]

#5	#1 OR #2 OR #3 OR #4
#6	“睡眠呼吸暂停综合征”［不加权:扩展］
#7	“睡眠呼吸暂停综合征”［常用字段:智能］OR “睡眠呼吸暂停综合症”［常用字段:智能］OR “低通气综合征”［常用字段:智能］OR “睡眠呼吸紊乱”［常用字段:智能］OR “睡眠呼吸暂停”［常用字段:智能］OR “睡眠呼吸障碍”［常用字段:智能］OR “睡眠呼吸不足”［常用字段:智能］OR “混合性睡眠呼吸暂停”［常用字段:智能］OR “阻塞性睡眠呼吸暂停”［常用字段:智能］OR “中枢性睡眠呼吸暂停”［常用字段:智能］OR “睡眠障碍性呼吸”［常用字段:智能］OR “鼾症”［常用字段:智能］OR “SAHS”［常用字段:智能］OR “SAS”［常用字段:智能］
#8	#6 OR #7
#9	“针灸疗法”［不加权:扩展］
#10	“针刺”［常用字段:智能］OR “针刺法”［常用字段:智能］OR “针刺治疗”［常用字段:智能］OR “针刺疗法”［常用字段:智能］OR “针刺临床”［常用字段:智能］OR “针灸”［常用字段:智能］OR “针灸法”［常用字段:智能］OR “针灸治疗”［常用字段:智能］OR “针灸疗法”［常用字段:智能］OR “针灸临床”［常用字段:智能］OR “针”［常用字段:智能］
#11	#9 OR #10
#12	#5 AND #8 AND #11
