# Supplementary material for: Efficacy and safety of acupuncture in the treatment of stroke complicated with sleep apnea syndrome: A systematic review and meta-analysis of randomized controlled trials
Source: Medicine (Baltimore). 2023 Apr 14;102(15):e33241. doi: 10.1097/MD.0000000000033241 (PMC10101308; doi:10.1097/MD.0000000000033241)

Supplemental Digital Content 2: Figure 1 that shows sensitivity analysis of total effective rate with the removal of Chen’s study.

	Experimental group		Control group			Risk Ratio
Study or Subgroup	Events	Total	Events	Total	Weight	M-H, Fixed, 95% CI
Chen 2020	39	42	32	42	0.0%	1.22 [1.01, 1.47]
Liu 2018	38	40	30	40	21.5%	1.27 [1.04, 1.54]
Lv 2017	34	39	24	40	17.0%	1.45 [1.10, 1.92]
Wang 2015	59	63	52	63	37.2%	1.13 [1.00, 1.29]
Zhang 2021	41	44	34	44	24.3%	1.21 [1.01, 1.44]
**Total (95% CI)**		**186**		**187**	**100.0%**	**1.23 [1.13, 1.35]**
Total events	172		140			

Heterogeneity: $Chi^2 = 3.04$, $df = 3$ ($P = 0.39$); $I^2 = 1\%$

Test for overall effect: $Z = 4.49$ ($P < 0.00001$)

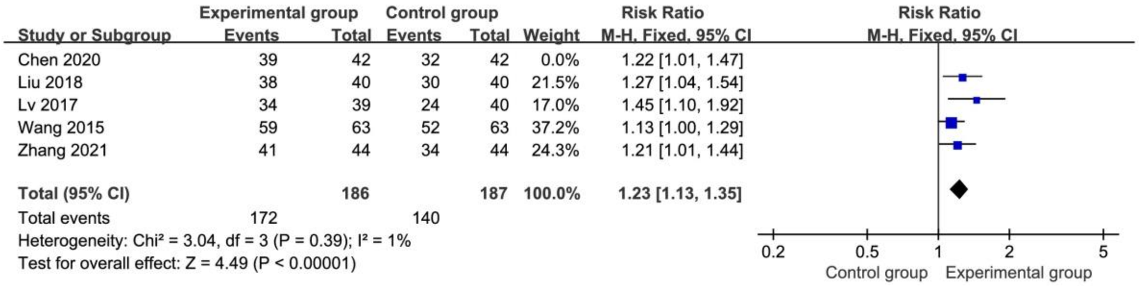

Supplemental Digital Content 3: Figure 2 that shows sensitivity analysis of total effective rate with the removal of Liu’s study.

	Experimental group		Control group			Risk Ratio
Study or Subgroup	Events	Total	Events	Total	Weight	M-H, Fixed, 95% CI
Chen 2020	39	42	32	42	22.6%	1.22 [1.01, 1.47]
Liu 2018	38	40	30	40	0.0%	1.27 [1.04, 1.54]
Lv 2017	34	39	24	40	16.7%	1.45 [1.10, 1.92]
Wang 2015	59	63	52	63	36.7%	1.13 [1.00, 1.29]
Zhang 2021	41	44	34	44	24.0%	1.21 [1.01, 1.44]
**Total (95% CI)**		**188**		**189**	**100.0%**	**1.22 [1.12, 1.34]**
Total events	173		142			

Heterogeneity: $Chi^2 = 2.76$, $df = 3$ ($P = 0.43$); $I^2 = 0\%$

Test for overall effect: $Z = 4.33$ ($P < 0.0001$)

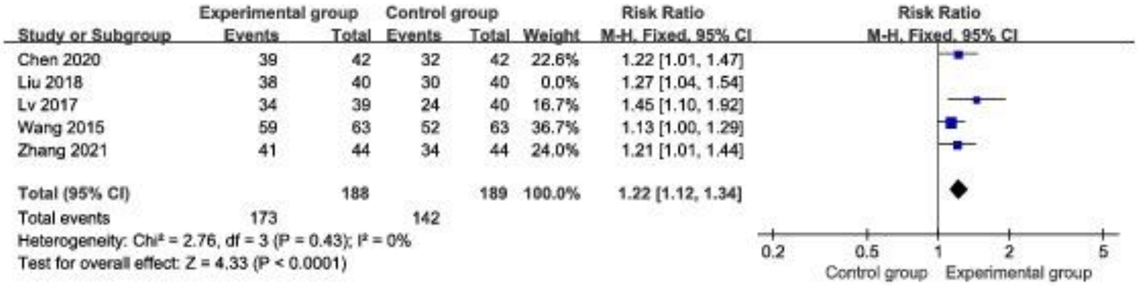

Supplemental Digital Content 4: Figure 3 that shows sensitivity analysis of total effective rate with the removal of Lv's study.

	Experimental group		Control group			Risk Ratio
Study or Subgroup	Events	Total	Events	Total	Weight	M-H, Fixed, 95% CI
Chen 2020	39	42	32	42	21.6%	1.22 [1.01, 1.47]
Liu 2018	38	40	30	40	20.3%	1.27 [1.04, 1.54]
Lv 2017	34	39	24	40	0.0%	1.45 [1.10, 1.92]
Wang 2015	59	63	52	63	35.1%	1.13 [1.00, 1.29]
Zhang 2021	41	44	34	44	23.0%	1.21 [1.01, 1.44]
**Total (95% CI)**		**189**		**189**	**100.0%**	**1.20 [1.10, 1.30]**
Total events	177		148			

Heterogeneity: $Chi^2 = 1.01$, $df = 3$ ($P = 0.80$); $I^2 = 0\%$

Test for overall effect: $Z = 4.19$ ($P < 0.0001$)

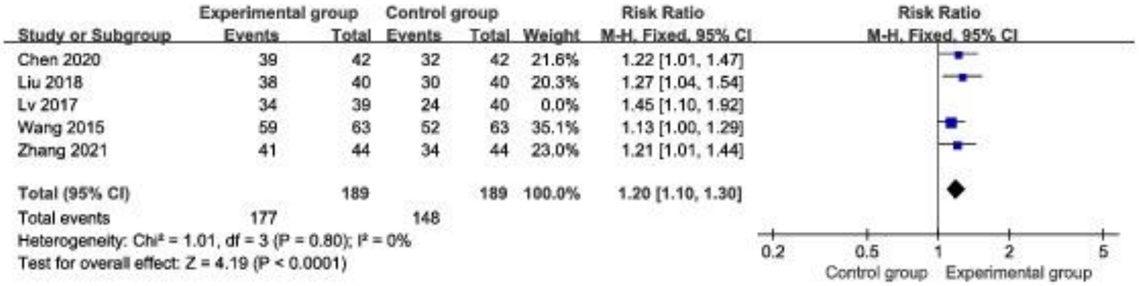

Supplemental Digital Content 5: Figure 4 that shows sensitivity analysis of total effective rate with the removal of Wang’s study.

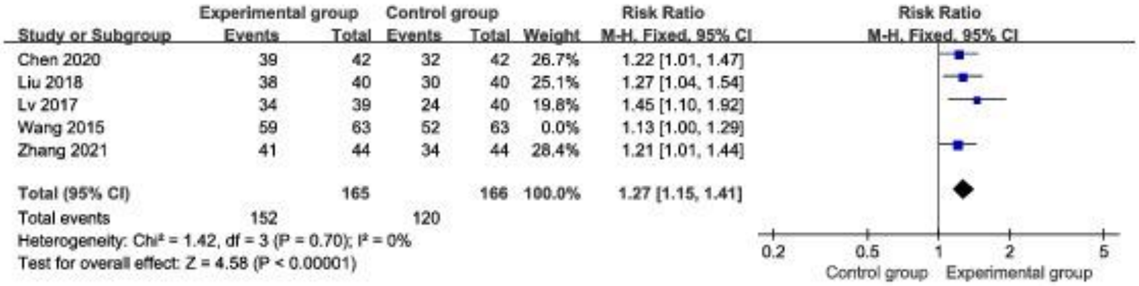

Supplemental Digital Content 6: Figure 5 that shows sensitivity analysis of total effective rate with the removal of Zhang’s study.

	Experimental group		Control group			Risk Ratio
Study or Subgroup	Events	Total	Events	Total	Weight	M-H, Fixed, 95% CI
Chen 2020	39	42	32	42	23.2%	1.22 [1.01, 1.47]
Liu 2018	38	40	30	40	21.8%	1.27 [1.04, 1.54]
Lv 2017	34	39	24	40	17.2%	1.45 [1.10, 1.92]
Wang 2015	59	63	52	63	37.8%	1.13 [1.00, 1.29]
Zhang 2021	41	44	34	44	0.0%	1.21 [1.01, 1.44]
**Total (95% CI)**		**184**		**185**	**100.0%**	**1.24 [1.13, 1.36]**
Total events	170		138			

Heterogeneity: Chi² = 3.05, df = 3 (P = 0.38); I² = 1%

Test for overall effect: Z = 4.49 (P < 0.00001)

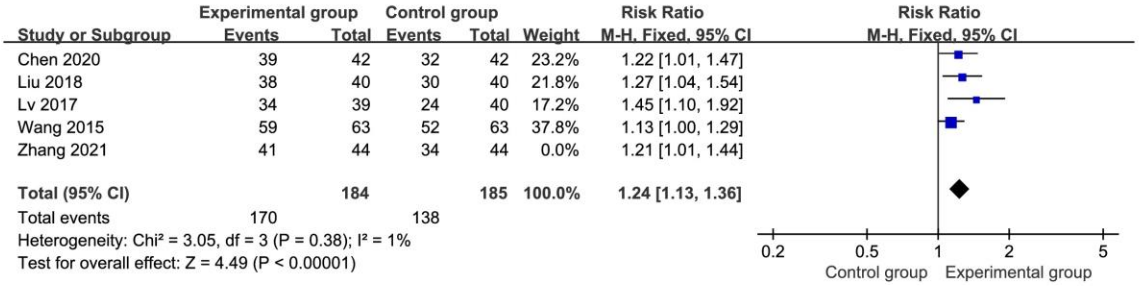

Supplement: Supplementary file 2 [file medi-102-e33241-s002.pdf]
