# Supplementary material for: Efficacy and safety of acupuncture in the treatment of stroke complicated with sleep apnea syndrome: A systematic review and meta-analysis of randomized controlled trials
Source: Medicine (Baltimore). 2023 Apr 14;102(15):e33241. doi: 10.1097/MD.0000000000033241 (PMC10101308; doi:10.1097/MD.0000000000033241)

Supplemental Digital Content 7: Figure 6 that shows sensitivity analysis of AHI with the removal of Chen's study.

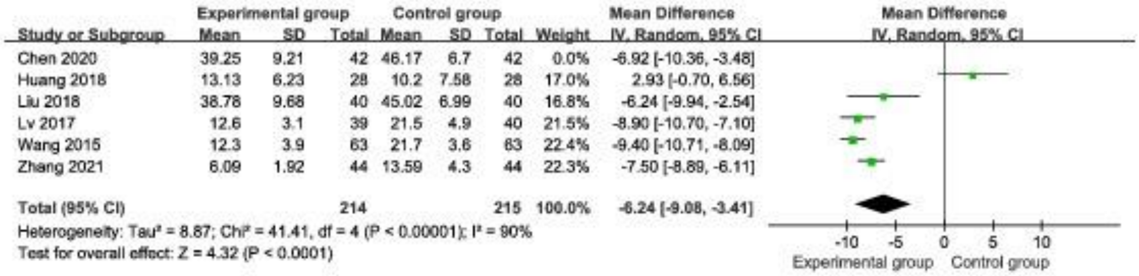

Supplemental Digital Content 8: Figure 7 that shows sensitivity analysis of AHI with the removal of Liu's study.

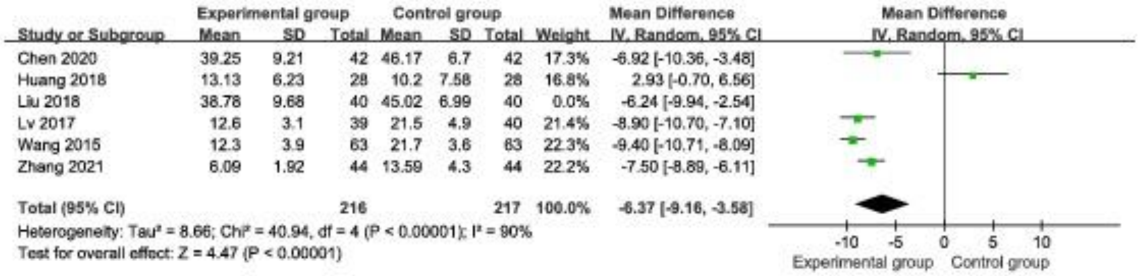

| | Experimental group | | | Control group | | | | Mean Difference |
|---|---|---|---|---|---|---|---|---|
| Study or Subgroup | Mean | SD | Total | Mean | SD | Total | Weight | IV, Random, 95% CI |
| Chen 2020 | 39.25 | 9.21 | 42 | 46.17 | 6.7 | 42 | 17.3% | -6.92 [-10.36, -3.48] |
| Huang 2018 | 13.13 | 6.23 | 28 | 10.2 | 7.58 | 28 | 16.8% | 2.93 [-0.70, 6.56] |
| Liu 2018 | 38.78 | 9.68 | 40 | 45.02 | 6.99 | 40 | 0.0% | -6.24 [-9.94, -2.54] |
| Lv 2017 | 12.6 | 3.1 | 39 | 21.5 | 4.9 | 40 | 21.4% | -8.90 [-10.70, -7.10] |
| Wang 2015 | 12.3 | 3.9 | 63 | 21.7 | 3.6 | 63 | 22.3% | -9.40 [-10.71, -8.09] |
| Zhang 2021 | 6.09 | 1.92 | 44 | 13.59 | 4.3 | 44 | 22.2% | -7.50 [-8.89, -6.11] |
| **Total (95% CI)** | | | **216** | | | **217** | **100.0%** | **-6.37 [-9.16, -3.58]** |

Heterogeneity: $Tau^2 = 8.66$; $Chi^2 = 40.94$, df = 4 ($P < 0.00001$); $I^2 = 90\%$

Test for overall effect: $Z = 4.47$ ($P < 0.00001$)

Supplemental Digital Content 9: Figure 8 that shows sensitivity analysis of AHI with the removal of Lv’s study.

| | Experimental group | | | Control group | | | | Mean Difference |
|---|---|---|---|---|---|---|---|---|
| Study or Subgroup | Mean | SD | Total | Mean | SD | Total | Weight | IV, Random, 95% CI |
| Chen 2020 | 39.25 | 9.21 | 42 | 46.17 | 6.7 | 42 | 18.5% | -6.92 [-10.36, -3.48] |
| Huang 2018 | 13.13 | 6.23 | 28 | 10.2 | 7.58 | 28 | 18.1% | 2.93 [-0.70, 6.56] |
| Liu 2018 | 38.78 | 9.68 | 40 | 45.02 | 6.99 | 40 | 17.9% | -6.24 [-9.94, -2.54] |
| Lv 2017 | 12.6 | 3.1 | 39 | 21.5 | 4.9 | 40 | 0.0% | -8.90 [-10.70, -7.10] |
| Wang 2015 | 12.3 | 3.9 | 63 | 21.7 | 3.6 | 63 | 22.8% | -9.40 [-10.71, -8.09] |
| Zhang 2021 | 6.09 | 1.92 | 44 | 13.59 | 4.3 | 44 | 22.7% | -7.50 [-8.89, -6.11] |
| Total (95% CI) | | | 217 | | | 217 | 100.0% | -5.72 [-8.87, -2.56] |

Heterogeneity: $Tau^2 = 10.91$; $Chi^2 = 40.18$, df = 4 ($P < 0.00001$); $I^2 = 90\%$

Test for overall effect: Z = 3.55 (P = 0.0004)

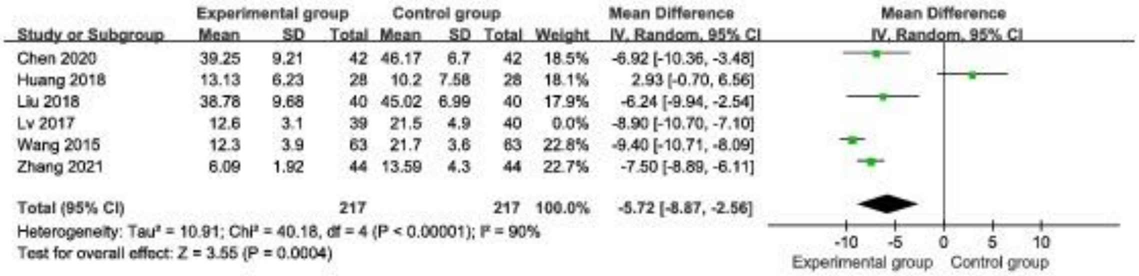

Supplemental Digital Content 10: Figure 9 that shows sensitivity analysis of AHI with the removal of Wang’s study.

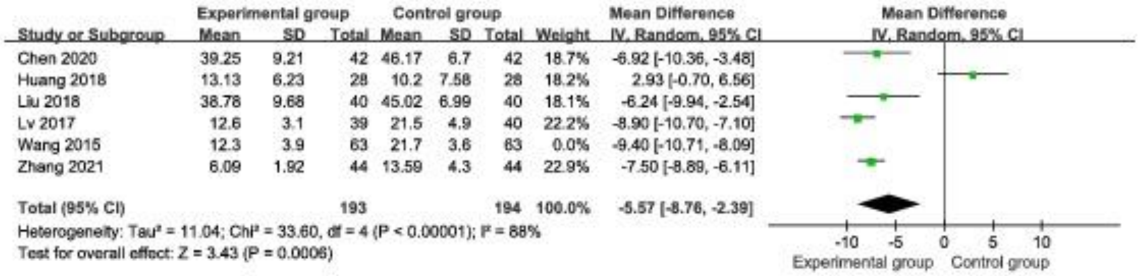

Supplemental Digital Content 11: Figure 10 that shows sensitivity analysis of AHI with the removal of Zhang's study.

| | Experimental group | | | Control group | | | | Mean Difference |
|---|---|---|---|---|---|---|---|---|
| Study or Subgroup | Mean | SD | Total | Mean | SD | Total | Weight | IV, Random, 95% CI |
| Chen 2020 | 39.25 | 9.21 | 42 | 46.17 | 6.7 | 42 | 18.9% | -6.92 [-10.36, -3.48] |
| Huang 2018 | 13.13 | 6.23 | 28 | 10.2 | 7.58 | 28 | 18.5% | 2.93 [-0.70, 6.56] |
| Liu 2018 | 38.78 | 9.68 | 40 | 45.02 | 6.99 | 40 | 18.4% | -6.24 [-9.94, -2.54] |
| Lv 2017 | 12.6 | 3.1 | 39 | 21.5 | 4.9 | 40 | 21.8% | -8.90 [-10.70, -7.10] |
| Wang 2015 | 12.3 | 3.9 | 63 | 21.7 | 3.6 | 63 | 22.5% | -9.40 [-10.71, -8.09] |
| Zhang 2021 | 6.09 | 1.92 | 44 | 13.59 | 4.3 | 44 | 0.0% | -7.50 [-8.89, -6.11] |
| Total (95% CI) | | | 212 | | | 213 | 100.0% | -5.96 [-9.43, -2.50] |

Heterogeneity: $Tau^2 = 13.49$; $Chi^2 = 41.33$, df = 4 ($P < 0.00001$); $I^2 = 90\%$

Test for overall effect: Z = 3.37 (P = 0.0007)

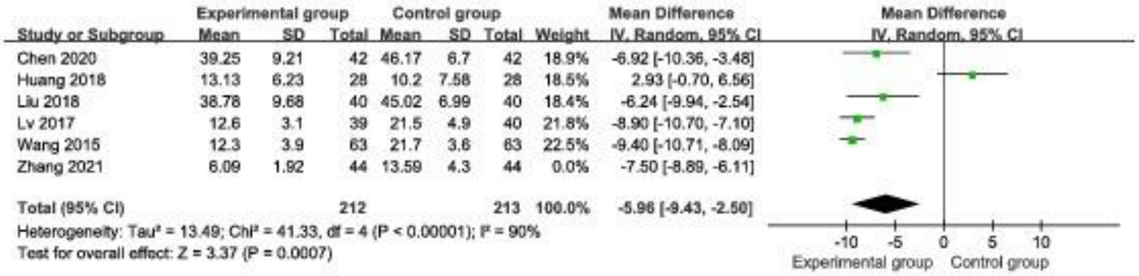

Supplement: Supplementary file 3 [file medi-102-e33241-s003.pdf]
