# Supplementary material for: Efficacy and safety of acupuncture in the treatment of stroke complicated with sleep apnea syndrome: A systematic review and meta-analysis of randomized controlled trials
Source: Medicine (Baltimore). 2023 Apr 14;102(15):e33241. doi: 10.1097/MD.0000000000033241 (PMC10101308; doi:10.1097/MD.0000000000033241)

Supplemental Digital Content 12: Figure 11 that shows sensitivity analysis of ESS score with the removal of Huang's study.

Study or Subgroup	Experimental group Mean	SD	Total	Control group Mean	SD	Total	Weight	Mean Difference IV, Random, 95% CI
Huang 2018	2.51	1.61	28	2.67	1.34	28	0.0%	-0.16 [-0.94, 0.62]
Liu 2018	5.21	0.63	40	6.1	0.72	40	35.4%	-0.89 [-1.19, -0.59]
Lv 2017	11.2	2	39	14.3	2.3	40	29.6%	-3.10 [-4.05, -2.15]
Wang 2015	11.8	1.2	63	14.1	0.9	63	35.0%	-2.30 [-2.67, -1.93]
**Total (95% CI)**			**142**			**143**	**100.0%**	**-2.04 [-3.26, -0.82]**

Heterogeneity: $Tau^2 = 1.07$; $Chi^2 = 45.00$, df = 2 (P < 0.00001); $I^2 = 96\%$
Test for overall effect: Z = 3.28 (P = 0.001)

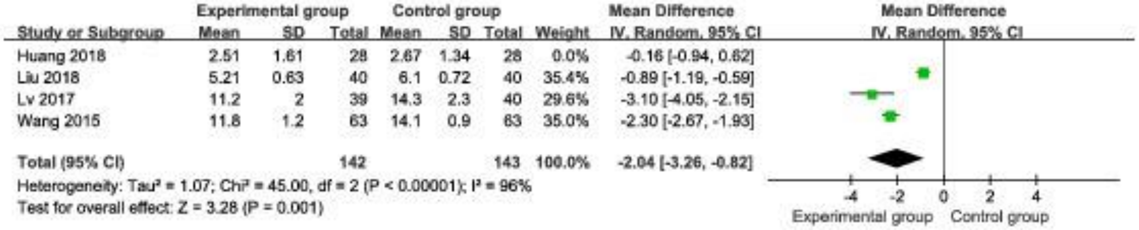

Supplemental Digital Content 13: Figure 12 that shows sensitivity analysis of ESS score with the removal of Liu's study.

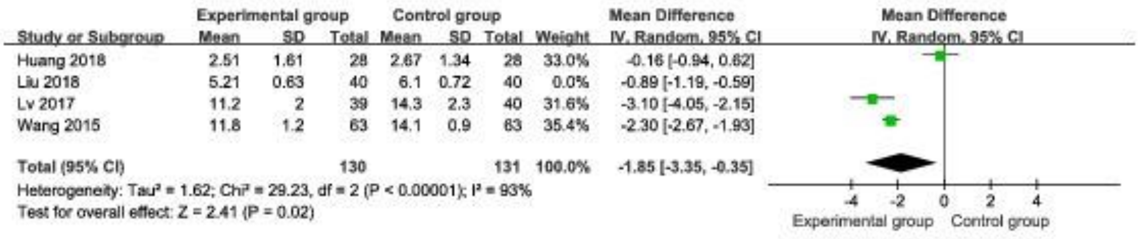

Supplemental Digital Content 14: Figure 13 that shows sensitivity analysis of ESS score with the removal of Lv's study.

	Experimental group			Control group				Mean Difference
Study or Subgroup	Mean	SD	Total	Mean	SD	Total	Weight	IV, Random, 95% CI
Huang 2018	2.51	1.61	28	2.67	1.34	28	30.7%	-0.16 [-0.94, 0.62]
Liu 2018	5.21	0.63	40	6.1	0.72	40	34.9%	-0.89 [-1.19, -0.59]
Lv 2017	11.2	2	39	14.3	2.3	40	0.0%	-3.10 [-4.05, -2.15]
Wang 2015	11.8	1.2	63	14.1	0.9	63	34.4%	-2.30 [-2.67, -1.93]
Total (95% CI)			131			131	100.0%	-1.15 [-2.30, -0.00]

Heterogeneity: $Tau^2 = 0.96$; $Chi^2 = 43.54$, df = 2 ($P < 0.00001$); $I^2 = 95\%$
Test for overall effect: Z = 1.96 (P = 0.05)

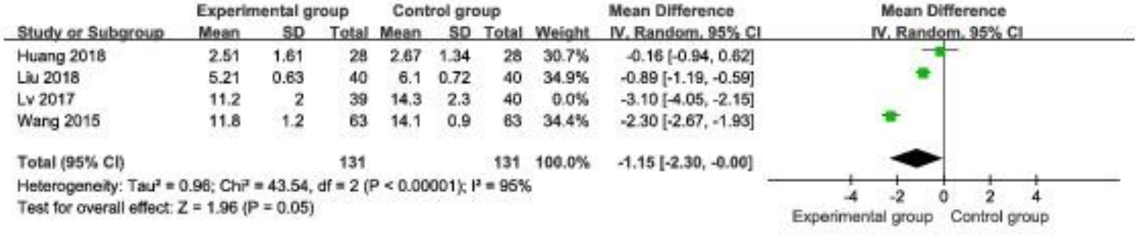

Supplemental Digital Content 15: Figure 14 that shows sensitivity analysis of ESS score with the removal of Wang's study.

	Experimental group			Control group				Mean Difference
Study or Subgroup	Mean	SD	Total	Mean	SD	Total	Weight	IV, Random, 95% CI
Huang 2018	2.51	1.61	28	2.67	1.34	28	32.7%	-0.16 [-0.94, 0.62]
Liu 2018	5.21	0.63	40	6.1	0.72	40	36.3%	-0.89 [-1.19, -0.59]
Lv 2017	11.2	2	39	14.3	2.3	40	31.0%	-3.10 [-4.05, -2.15]
Wang 2015	11.8	1.2	63	14.1	0.9	63	0.0%	-2.30 [-2.67, -1.93]
Total (95% CI)			107			108	100.0%	-1.34 [-2.64, -0.03]

Heterogeneity: $Tau^2 = 1.20$; $Chi^2 = 23.78$, df = 2 ($P < 0.00001$); $I^2 = 92\%$
Test for overall effect: Z = 2.00 (P = 0.05)

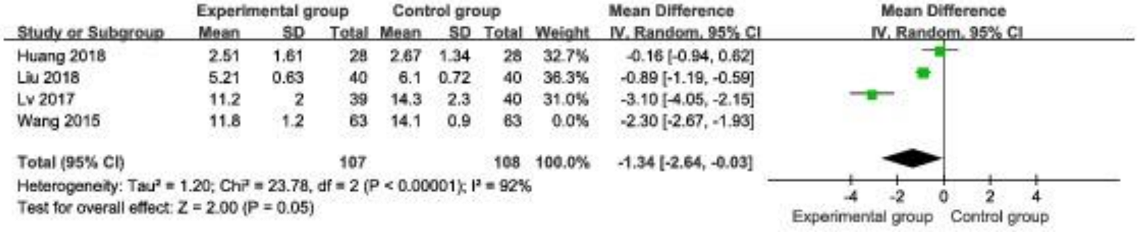

Supplement: Supplementary file 4 [file medi-102-e33241-s004.pdf]
