# Supplementary material for: Efficacy and safety of acupuncture in the treatment of stroke complicated with sleep apnea syndrome: A systematic review and meta-analysis of randomized controlled trials
Source: Medicine (Baltimore). 2023 Apr 14;102(15):e33241. doi: 10.1097/MD.0000000000033241 (PMC10101308; doi:10.1097/MD.0000000000033241)

Supplemental Digital Content 17: Figure 16 that shows sensitivity analysis of SaO_2min with the removal of Zhang's study.

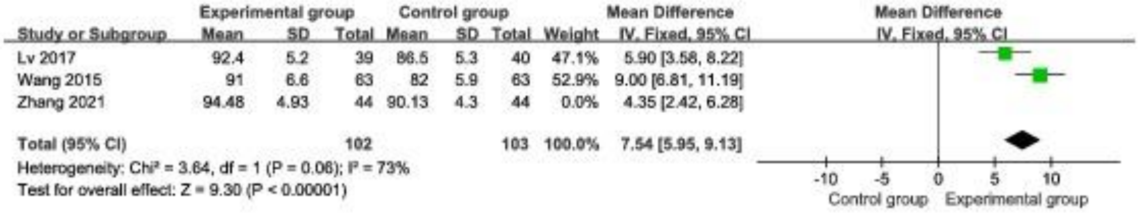

Supplement: Supplementary file 6 [file medi-102-e33241-s006.pdf]
