# Supplementary material for: Efficacy and safety of acupuncture in the treatment of stroke complicated with sleep apnea syndrome: A systematic review and meta-analysis of randomized controlled trials
Source: Medicine (Baltimore). 2023 Apr 14;102(15):e33241. doi: 10.1097/MD.0000000000033241 (PMC10101308; doi:10.1097/MD.0000000000033241)

Supplemental Digital Content 18: Figure 17 that shows sensitivity analysis of longest duration of apnea with the removal of Chen's study.

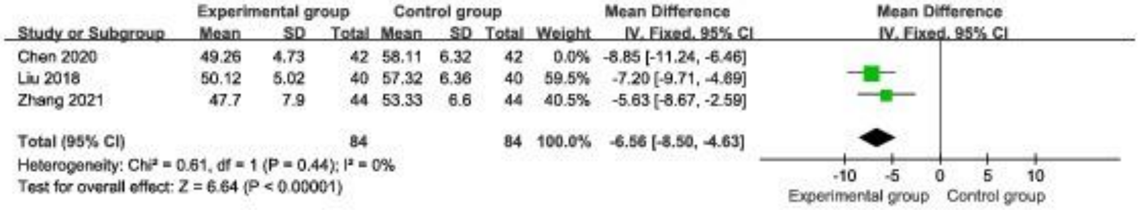

Supplemental Digital Content 19: Figure 18 that shows sensitivity analysis of longest duration of apnea with the removal of Liu's study.

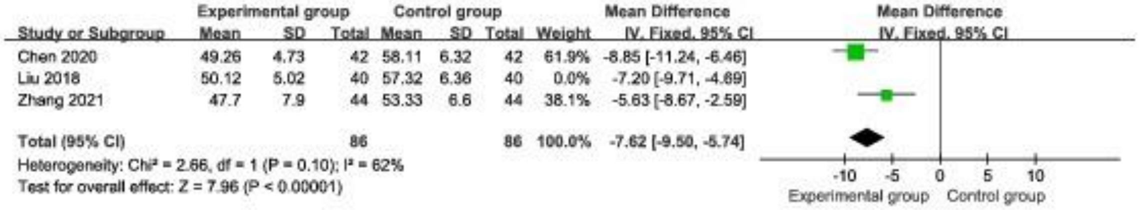

	Experimental group			Control group				Mean Difference
Study or Subgroup	Mean	SD	Total	Mean	SD	Total	Weight	IV, Fixed, 95% CI
Chen 2020	49.26	4.73	42	58.11	6.32	42	61.9%	-8.85 [-11.24, -6.46]
Liu 2018	50.12	5.02	40	57.32	6.36	40	0.0%	-7.20 [-9.71, -4.69]
Zhang 2021	47.7	7.9	44	53.33	6.6	44	38.1%	-5.63 [-8.67, -2.59]
Total (95% CI)			86			86	100.0%	-7.62 [-9.50, -5.74]

Heterogeneity: Chi² = 2.66, df = 1 (P = 0.10); I² = 62%
Test for overall effect: Z = 7.96 (P < 0.00001)

Supplemental Digital Content 20: Figure 19 that shows sensitivity analysis of longest duration of apnea with the removal of Zhang’s study.

	Experimental group			Control group				Mean Difference
Study or Subgroup	Mean	SD	Total	Mean	SD	Total	Weight	IV, Fixed, 95% CI
Chen 2020	49.26	4.73	42	58.11	6.32	42	52.5%	-8.85 [-11.24, -6.46]
Liu 2018	50.12	5.02	40	57.32	6.36	40	47.5%	-7.20 [-9.71, -4.69]
Zhang 2021	47.7	7.9	44	53.33	6.6	44	0.0%	-5.63 [-8.67, -2.59]
Total (95% CI)			82			82	100.0%	-8.07 [-9.80, -6.34]

Heterogeneity: $Chi^2 = 0.87$, $df = 1$ ($P = 0.35$); $I^2 = 0\%$
Test for overall effect: $Z = 9.14$ ($P < 0.00001$)

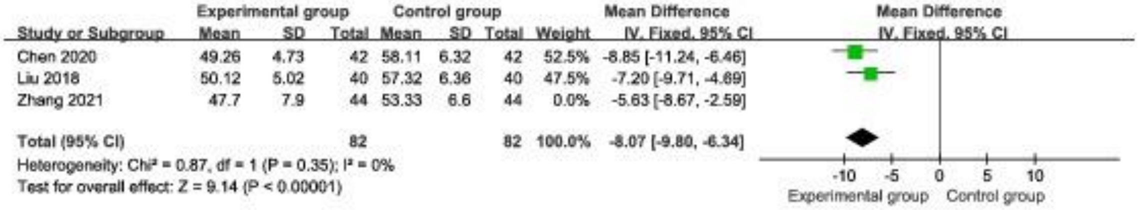

Supplement: Supplementary file 7 [file medi-102-e33241-s007.pdf]
